# Melting temperature mapping method using imperfect-match linear long probes

**DOI:** 10.1038/s41598-024-60987-7

**Published:** 2024-05-14

**Authors:** Shinya Ootsuki, Hideki Niimi, Tomohiro Ueno, Masashi Mori, Homare Tabata, Hiroshi Minami, Isao Kitajima

**Affiliations:** 1 Clinical Laboratory and Transfusion Medicine & Cell Therapy Center, Toyama University Hospital, 2630 Sugitani, Toyama, 930-0194 Japan; 2 Laboratory for Clinical Investigation, Osaka University Hospital, Osaka, 565-0871 Japan; 3 Research Institute for Bioresources and Biotechnology, Ishikawa Prefectural University, Ishikawa, 921-8836 Japan; 4 Life Science Center, Hokkaido Mitsui Chemicals, Inc., Hokkaido, 073-0138 Japan; 5 Administrative Office, University of Toyama, Toyama, 930-8555 Japan

**Keywords:** PCR-based techniques, Bacterial infection

## Abstract

Identifying pathogenic microorganisms as early as possible is critical for selecting the appropriate antimicrobial therapy in infected patients. We previously reported the development of the Tm mapping method for identifying a broad range of pathogenic bacteria within 3 h of blood collection. However, the Tm mapping identification requires an analytical instrument with a tube-to-tube variation of no more than 0.1 °C, so we can only use a few instruments that have such high thermal accuracy. To address the problem, we developed the improved Tm mapping method using imperfect-match linear long quenching probes (IMLL Q-probes). Using IMLL Q-probes, almost all commercially available analytical instruments can be used for the Tm mapping method. Some bacterial species cannot be narrowed down to one species, but they can at least be narrowed down to the genus level. The Tm mapping method using IMLL Q-probes is useful for deciding on antimicrobial therapy in infected patients.

## Introduction

Sepsis is a syndrome characterized by whole-body inflammation due to infection and is the primary cause of morbidity and mortality in hospitalized patients^1^. A definitive diagnosis of sepsis requires proper identification of the causative microorganism. However, as current pathogen-identification methods using microbial cultures require several days, empirically selected antimicrobial agents are often administered until the pathogenic microbes are identified^2,3^. As long as microbial cultures are used, it is difficult to establish a rapid system because the speed of detection depends on the growth rate of the bacterial species. In this regard, even mass spectrometry-based identification, which at present also require microbial culture, is no exception^4,5^.

Identifying pathogenic microorganisms as early as possible is critical for selecting the appropriate antimicrobial therapy and obtaining a favorable outcome in infected patients^6–8^. We previously reported the development of a novel rapid, easy and cost-effective method known as the melting temperature (Tm) mapping method for identifying a broad range of pathogenic bacteria within three hours of whole blood collection^9^. However, as a weak point, the Tm mapping identification requires a measurement error of no more than 0.1 °C among polymerase chain reaction (PCR) tubes within the same trial (tube-to-tube variation), so we can only use optimal analytical instruments, such as the RotorGeneQ (QIAGEN, Germany) or LightCycler® Nano (Roche Applied Science, Germany). This is a major obstacle to the popularization of the Tm mapping method.

In an attempt to address the problem above, we developed an improved Tm mapping method using imperfect-match linear long probes. Using the long probes, the Tm mapping method can generate a wider variation range of Tm values, so almost all commercially available analytical instruments can be used for the Tm mapping method.

## Results

### Workflow of the Tm mapping method using imperfect-match linear long quenching probes (IMLL Q-probes)

**Figure 1:**
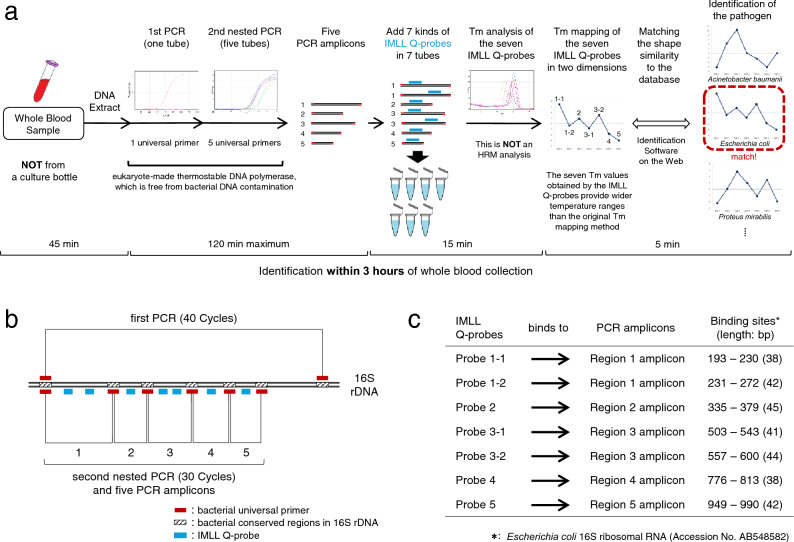
Workflow of the melting temperature mapping method using imperfect-match linear long quenching probes (IMLL Q-probes). (**a**) Workflow of the Tm mapping method using IMLL Q-probes for identifying unknown pathogenic bacteria within three hours of whole blood collection. (**b**) The strategy for the primer designs is shown. Nested PCR is performed using five bacterial universal primer sets, seven IMLL Q-probes are additionally used for PCR amplicons respectively, and then seven Tm values are obtained. (**c**) Binding sites of each IMLL Q-probes.

The workflow of the Tm mapping method using IMLL Q-probes to identify unknown pathogens, which we developed, is shown in Fig. 1a. This method identifies the predominant (usually pathogenic) bacteria in a clinical sample within three hours of whole blood collection. Of note, unlike the current Tm mapping method^9^, the improved Tm mapping method using IMLL Q-probes can be used with almost all commercially available real-time PCR instruments.

This method consists of four major steps. First, bacterial DNA are extracted directly from a clinical sample (2 mL of a whole blood sample, etc.) as a template for PCR. Step two involves nested PCR using five bacterial universal primer sets (one primer set per tube in the second PCR); these primers can amplify almost all species of bacteria (Fig. 1b) but does not amplify fungi nor human DNA. To achieve accuracy in this PCR step, we developed a eukaryote-made thermostable DNA polymerase that is free from bacterial DNA contamination^10^. The eukaryote-made thermostable DNA polymerase is a recombinant polymerase manufactured using eukaryotic (yeast) host cells. Employing this DNA polymerase, sensitive and reliable detection of bacteria without false-positive results is feasible, thereby making it possible for PCR to identify bacterial isolates directly from patient samples. The nested PCR procedure is performed, and five (or fewer) PCR amplicons are obtained. In step three, Region 1 and 3 PCR amplicons are divided into two parts, and then a total of seven PCR amplicons are mixed with seven kinds of IMLL Q-probes (Fig. 1a–c). Specifically, Probe 1–1 is mixed in a PCR tube containing Region 1 amplicon, Probe 1–2 is mixed in a PCR tube containing Region 1 amplicon, Probe 2 is mixed in a PCR tube containing Region 2 amplicon, etc. Seven Tm values are then acquired by analyzing the seven IMLL Q-probes. Step four involves mapping the seven Tm values in two dimensions (see Fig. 1a). The plot creates a unique species-specific shape known as the Tm mapping shape. Of note: this is *not* a high-resolution melting-curve (HRM) analysis, and only the Tm values are recorded. By comparing the Tm mapping shape to the shapes in the database, the bacterial isolates can be rapidly identified.

### Strategies in designing IMLL Q-probes

In order for the improved Tm mapping method to work on almost all real-time PCR instruments, we designed the IMLL Q-probes. Four points are important to consider when designing IMLL Q-probes. First, strive to make it possible to obtain a wider variation range of Tm values for different species of bacteria. Second, design probes long enough (around 40-mer) to bind almost all species of bacteria, even those with many probe-target mismatches. Third, design long probes that do not have secondary structures but rather have linear structures to prevent self-quenching. Finally, design probes so as to obtain a wide variety of Tm values for each bacterial species and to identify various bacterial species.

We then designed the IMLL Q-probes for the Tm mapping method to generate a wider variation range of Tm values than the current Tm mapping method. As a result, using IMLL Q-probes, the ranges of Tm values of 71 bacterial species registered in the database were more than 20 °C wide (probe 1–1: 49.32–72.42 °C, probe 1–2: 47.44–67.57 °C, probe 2: 43.29–67.30 °C, probe 3–1: 47.17–68.61 °C, probe 3–2: 43.95–68.55 °C, probe 4: 44.13–66.54 °C, probe 5: 43.19–64.23 °C) (Supplemental Fig. S1). In contrast, the ranges of Tm values of the current Tm mapping method were less than 4 °C wide.

We designed seven long probes that could bind positions with many probe-target mismatches (Supplemental Table S1). A sufficient probe length creates more hydrogen bonds than mismatches, so the IMLL Q-probe can bind many bacterial species with different base sequences. Furthermore, using the delta G value, these long probes were strictly designed to prevent a secondary structure in the probes themselves from forming. The delta G value of any self-dimers, hairpins, and heterodimers should be weaker (more positive) than − 9.0 kcal/mol. Positive numbers indicate that the actual secondary structure shown will not form at all. We named these long quenching probes imperfect-match linear long quenching probes (IMLL Q-probes).

### Construction of the Tm mapping database using IMLL Q-probes

**Figure 2:**
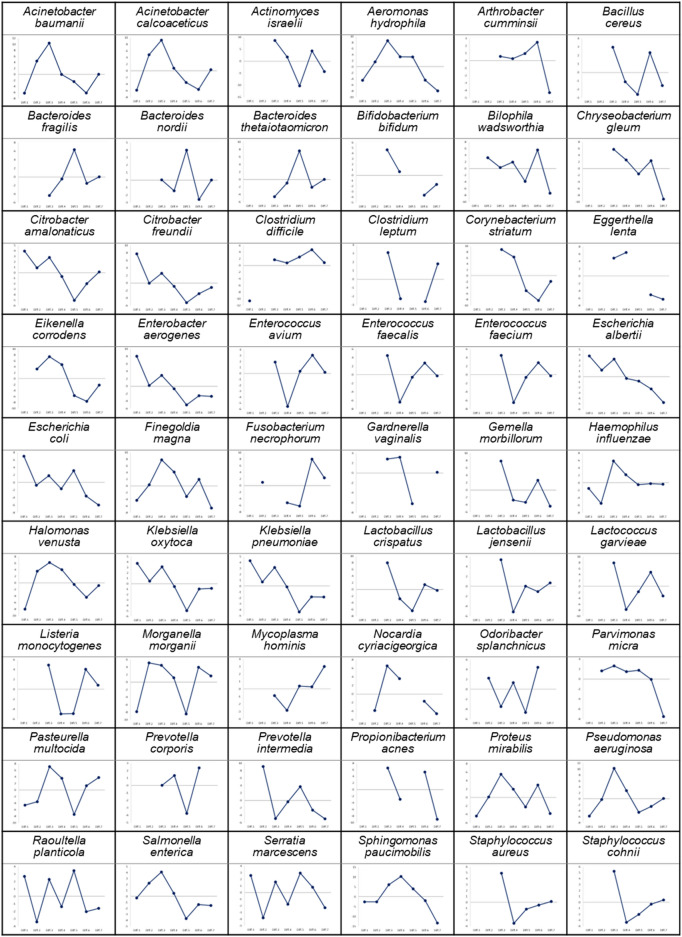

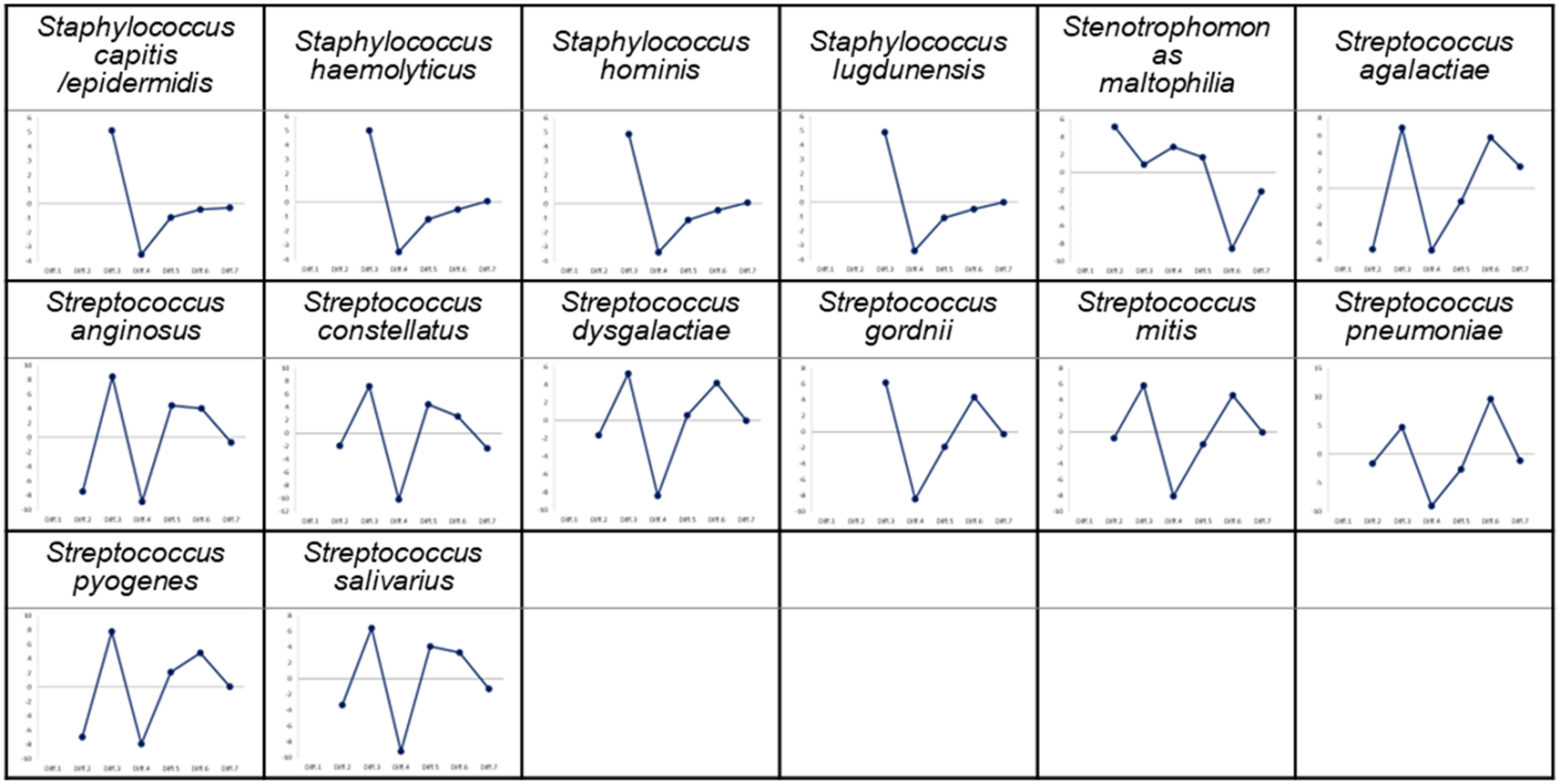
The Tm mapping shapes (IMLL Q-probes) of the 68 species of bacteria registered in the database. The horizontal line drawn in each Tm mapping shape is the average of the seven or fewer Tm values using IMLL Q-probes.

Using the mean of triplicate Tm value measurements with IMLL Q-probes, we constructed a preliminary database with the Tm mapping shapes of 68 species (Fig. 2). These species of bacteria were obtained from clinical samples and then Sanger sequenced and identified at the species level. The Tm mapping database is scalable and can be easily modified and updated. The individual Tm mapping shapes in the database show unique shapes reflecting the number and position of probe-target mismatches on IMLL Q-probe hybridizations of each bacterial species. Some Tm mapping shapes are missing data points; this is due to the fact that such Tm values cannot be obtained because the IMLL Q-probes do not bind to their target regions. To identify the bacterial isolate, the identification software program narrows the scope of its search to bacteria in the database with the same pattern as the IMLL Q-probe binding while also comparing the Tm mapping shapes. The pattern of the IMLL Q-probe binding is therefore also a characteristic of the bacteria.

Using the current protocols, the limits of identification are as follows: *Escherichia coli* = 5 CFU/PCR tube (250 CFU/mL), *Staphylococcus aureus* = 5 CFU/PCR tube (250 CFU/mL). The limits of identification were determined to be the final log_2_ dilution of the template in which the Tm mapping result was correct.

### Assessment of the accuracy of the Tm mapping method using IMLL Q-probes

To assess the accuracy of the Tm mapping method using IMLL Q-probes, we first performed blind tests using the 68 species of bacterial DNA registered in the database. Concealing the name of the bacteria, we tried to identify the bacterial DNA using the Rotor-Gene Q instrument (tube-to-tube variation ≤  ± 0.1 °C). Of the 68 bacterial species, 62 Tm mapping results matched the pre-sequenced bacterial DNA by Sanger sequencing, but 6 Tm mapping results were unable to be narrowed down to a single bacterial species. The Tm mapping method using IMLL Q-probes was unable to distinguish between *Enterococcus faecalis* and *Enterococcus faecium* or between *Staphylococcus aureus*, *Staphylococcus hemolyticus*, *Staphylococcus hominis* and *Staphylococcus lugdunensis*. The mean difference value was 0.242, with a range of 0.09–0.30 (standard deviation = 0.08).

Next, to confirm the ability to identify bacteria depending on real-time PCR instruments, we assess the Tm mapping shape similarity among 68 species of bacteria in the database (Table 1). We previously defined the difference value as the difference between the Tm mapping shape and that observed in the database^9^. The closer the difference value is to 0, the more similar the Tm mapping shape is to the shape of the bacteria registered in the database. If the tube-to-tube variation of the real-time PCR instrument is ≤  ± 0.1 °C, then the measurement error in the difference values would be ≤ 0.28, as calculated by the difference value formula:$\sqrt {(7 \times } \left( { \pm 0.1} \right)^{2} )$. In the same way, if the tube-to-tube variation of the real-time PCR instrument is ≤  ± 0.2 °C, ≤  ± 0.3 °C, ≤  ± 0.4 °C, ≤  ± 0.5 °C, or ≤  ± 0.6 °C, then the measurement error in the difference values would be ≤ 0.53, ≤ 0.80, ≤ 1.06, ≤ 1.33, and ≤ 1.59, respectively (Table 2). The tube-to-tube variation of almost all commercially available real-time PCR instruments is ≤  ± 0.5 °C, resulting in a difference value measurement error of ≤ 1.33. Based on the data obtained using the IMLL Q-probes, 60 of the 68 bacterial species in the database do not interfere with each other if the difference value measurement error is ≤ 1.33 (see Tables 1 and 2). Therefore, with almost all commercially available real-time PCR instruments, the Tm mapping method using IMLL Q-probes was able to identify at least 60 out of 68 bacteria at the species level; the remaining 8 Tm mapping results were matched at the genus level but were unable to be narrowed down to a single bacterial species.

**Table 1:** Tm mapping shape similarity among 68 species of bacteria in the database.

Bacteria in the database	The number of bacteria							Most similar bacteria (D) in database
	0 ≤ D ≤ 0.28	0.28 < D ≤ 0.53	0.53 < D ≤ 0.80	0.80 < D ≤ 1.06	1.06 < D ≤ 1.33	1.33 < D ≤ 1.59	1.59 < D	
*Acinetobacter baumanii*	0	0	0	0	0	0	**67**	*A. calcoaceticus* (1.99)
*Acinetobacter calcoaceticus*	0	0	0	0	0	0	**67**	*A. baumanii* (1.99)
*Actinomyces israelii*	0	0	0	0	0	0	**67**	*C. striatum* (6.15)
*Aeromonas hydrophilia*	0	0	0	0	0	0	**67**	*P. aeruginosa* (8.56)
*Arthrobacter cumminsii*	0	0	0	0	0	0	**67**	*P. micra* (4.48)
*Bacillus cereus*	0	0	0	0	0	0	**67**	*S. capitis/epidermidis* (4.46)
*Bacteroides fragilis*	0	0	0	0	0	**1**	**66**	*B. thetaiotaomicron* (1.48)
*Bacteroides nordii*	0	0	0	0	0	0	**67**	*B. fragilis* (5.16)
*Bacteroides thetaiotaomicron*	0	0	0	0	0	**1**	**66**	*B. fragilis* (1.48)
*Bifidobacterium bifidum*	0	0	0	0	0	0	**67**	*N. cyriacigeorgica* (4.54)
*Bilophila wadsworthia*	0	0	0	0	0	0	**67**	*P. micra* (8.45)
*Chryseobacterium gleum*	0	0	0	0	0	0	**67**	*P. micra* (5.15)
*Citrobacter amalonaticus*	0	0	0	0	0	**2**	**65**	*K. pneumoniae* (1.52)
*Citrobacter freundii*	0	0	0	0	0	0	**67**	*E. aerogenes* (1.63)
*Clostridium difficile*	0	0	0	0	0	0	**67**	*H. influenzae* (11.48)
*Clostridium leptum*	0	0	0	0	0	0	**67**	*N. cyriacigeorgica* (9.25)
*Corynebacterium striatum*	0	0	0	0	0	0	**67**	*S. maltophilia* (10.74)
*Eggerthella lenta*	0	0	0	0	0	0	**67**	*N. cyriacigeorgica* (4.69)
*Eikenella corrodens*	0	0	0	0	0	0	**67**	*S. maltophilia* (10.24)
*Enterobacter aerogenes*	0	0	0	0	0	0	**67**	*K. pneumoniae* (2.94)
*Enterococcus avium*	0	0	0	0	0	0	**67**	*E. casseliflavus* (2.74)
*Enterococcus faecalis*	**1**	0	0	0	0	0	**66**	*E. faecium* (0.11)
*Enterococcus faecium*	**1**	0	0	0	0	0	**66**	*E. faecalis* (0.21)
*Escherichia albertii*	0	0	0	0	0	0	**67**	*K. pneumoniae* (4.65)
*Escherichia coli*	0	0	0	0	0	0	**67**	*E. albertii* (6.09)
*Finegoldia magna*	0	0	0	0	0	0	**67**	*P. mirabilis* (4.44)
*Fusobacterium nucleatum*	0	0	0	0	0	0	**67**	None
*Gardnerella vaginalis*	0	0	0	0	0	0	**67**	*C. striatum* (5.88)
*Gemella morbillorum*	0	0	0	0	0	0	**67**	*L. crispatus* (5.26)
*Haemophilus influenzae*	0	0	0	0	0	0	**67**	*P. mirabilis* (8.07)
*Halomonas venusta*	0	0	0	0	0	0	**67**	*A. calcoaceticus* (6.14)
*Klebsiella oxytoca*	0	0	0	0	0	**1**	**66**	*C. amalonaticus* (1.55)
*Klebsiella pneumoniae*	0	0	0	0	0	**1**	**66**	*C. amalonaticus* (1.52)
*Lactobacillus crispatus*	0	0	0	0	0	0	**67**	*L. monocytogenes* (4.96)
*Lactobacillus jensenii*	0	0	0	0	0	0	**67**	*S. aureus* (2.22)
*Lactococcus garvieae*	0	0	0	0	0	0	**67**	*S. gordonii* (3.49)
*Listeria monocytogenes*	0	0	0	0	0	0	**67**	*E. faecium* (4.86)
*Morganella morganii*	0	0	0	0	0	0	**67**	*P. mirabilis* (10.39)
*Mycoplasma hominis*	0	0	0	0	0	0	**67**	*E. avium* (5.54)
*Nocardia cyriacigeorgica*	0	0	0	0	0	0	**67**	None
*Odoribacter splanchnicus*	0	0	0	0	0	0	**67**	None
*Parvimonas micra*	0	0	0	0	0	0	**67**	*A. cumminsii* (5.07)
*Pasteurella multocida*	0	0	0	0	0	0	**67**	*P. aeruginosa* (7.71)
*Prevotella corporis*	0	0	0	0	0	0	**67**	*B. nordii* (9.60)
*Prevotella intermedia*	0	0	0	0	0	0	**67**	*S. maltophilia* (10.36)
*Propionibacterium acnes*	0	0	0	0	0	0	**67**	*N. cyriacigeorgica* (10.28)
*Proteus mirabilis*	0	0	0	0	0	0	**67**	*F. magna* (4.44)
*Pseudomonas aeruginosa*	0	0	0	0	0	0	**67**	*A. calcoaceticus* (6.41)
*Raoultella planticola*	0	0	0	0	0	0	**67**	*S. marcescens* (3.09)
*Salmonella enterica*	0	0	0	0	0	0	**67**	*K. pneumoniae* (4.25)
*Serratia marcescens*	0	0	0	0	0	0	**67**	*R. planticola* (3.09)
*Sphingomonas paucimobilis*	0	0	0	0	0	0	**67**	*A. hydrophila* (10.59)
*Staphylococcus aureus*	**3**	0	**1**	**1**	0	0	**62**	*S. hemolyticus* (0.16)
*Staphylococcus capitis/epidermidis*	0	**1**	**3**	0	0	**1**	**62**	*S. lugdunensis* (0.48)
*Staphylococcus cohnii*	0	0	0	**3**	**1**	**1**	**62**	*S. haemolyticus* (0.96)
*Staphylococcus hemolyticus*	**3**	0	**1**	**1**	0	0	**62**	*S. aureus* (0.16)
*Staphylococcus hominis*	**3**	0	**1**	**1**	0	0	**62**	*S. lugdunensis* (0.10)
*Staphylococcus lugdunensis*	**3**	**1**	0	0	**1**	0	**62**	*S. hominis* (0.10)
*Stenotrophomonas maltophilia*	0	0	0	0	0	0	**67**	*C. striatum* (10.74)
*Streptococcus agalactiae*	0	0	0	0	0	0	**67**	*S. pyogenes* (4.59)
*Streptococcus anginosus*	0	0	0	0	0	0	**67**	*S. pyogenes* (2.85)
*Streptococcus constellatus*	0	0	0	0	0	0	**67**	*S. salivarius* (2.27)
*Streptococcus dysgalactiae*	0	0	0	0	0	0	**67**	*S. mitis* (2.45)
*Streptococcus gordnii*	0	0	0	0	0	0	**67**	*E. faecium* (3.24)
*Streptococcus mitis*	0	0	0	0	0	0	**67**	*S. dysgalactiae* (2.45)
*Streptococcus pneumoniae*	0	0	0	0	0	0	**67**	*S. mitis* (5.52)
*Streptococcus pyogenes*	0	0	0	0	0	0	**67**	*S. anginosus* (2.85)
*Streptococcus salivarius*	0	0	0	0	0	0	**67**	*S. constellatus* (2.27)

Non-zero numbers are in [bold].

D, difference value.

**Table 2:** Bacterial species that are indistinguishable using various instruments.

Tube to tube variation of the instrument	The measurement error of Difference Value (D)	Indistinguishable bacterial species registered in the database, which cannot be narrowed down to one species
± 0.1 °C	0.0 ≤ D ≤ 0.28	[*Enterococcus faecalis* and *Enterococcus faecium*] [*Staphylococcus aureus*, *Staphylococcus hemolyticus*, *Staphylococcus hominis* and *Staphylococcus lugdunensis*]
± 0.2 °C	0.0 ≤ D ≤ 0.53	[*Enterococcus faecalis* and *Enterococcus faecium*] [*Staphylococcus aureus*, *Staphylococcus hemolyticus*, *Staphylococcus hominis*, *Staphylococcus lugdunensis* and *Staphylococcus capitis/epidermidis*]
± 0.3 °C	0.0 ≤ D ≤ 0.80	[*Enterococcus faecalis* and *Enterococcus faecium*] [*Staphylococcus aureus*, *Staphylococcus hemolyticus*, *Staphylococcus hominis*, *Staphylococcus lugdunensis* and *Staphylococcus capitis/epidermidis*]
± 0.4 °C	0.0 ≤ D ≤ 1.06	[*Enterococcus faecalis* and *Enterococcus faecium*] [*Staphylococcus aureus*, *Staphylococcus hemolyticus*, *Staphylococcus hominis*, *Staphylococcus lugdunensis,* *Staphylococcus capitis/epidermidis* and *Staphylococcus cohnii*]
± 0.5 °C	0.0 ≤ D ≤ 1.33	[*Enterococcus faecalis* and *Enterococcus faecium*] [*Staphylococcus aureus*, *Staphylococcus hemolyticus*, *Staphylococcus hominis*, *Staphylococcus lugdunensis,* *Staphylococcus capitis/epidermidis* and *Staphylococcus cohnii*]
± 0.6 °C	0.0 ≤ D ≤ 1.59	[*Enterococcus faecalis* and *Enterococcus faecium*] [*Staphylococcus aureus*, *Staphylococcus hemolyticus*, *Staphylococcus hominis*, *Staphylococcus lugdunensis,* *Staphylococcus capitis/epidermidis* and *Staphylococcus cohnii*] [*Bacteroides fragilis* and *Bacteroides thetaiotaomicron*] [*Citrobacter amalonaticus*, *Klebsiella oxytoca* and *Klebsiella pneumoniae*]

Finally, using 18 whole blood samples collected from patients with sepsis, we compared the accuracy of the Tm mapping method using IMLL Q-probes with the LightCycler 480 instrument (tube-to-tube variation ≤  ± 0.4 °C) with that of the conventional culture method. The individual Tm mapping results using IMLL Q-probes compared with the culture or sequencing results are shown in Table 3. If the Tm mapping result did not match the culture result, we checked it again using the Sanger sequencing method (Supplementary Data). As a negative control, no bacteria were detected in healthy whole blood using either Tm mapping method or conventional culture method. Of a total of 18 Tm mapping results, 13 Tm mapping results were a match with the culture or sequencing results at the species level, but concerning *Enterococcus faecalis* or genus *Staphylococcus*, Tm mapping results were unable to be narrowed down to a single bacterial species. For example, in patient No. 16, the Tm mapping result was genus *Staphylococcus* (*Staphylococcus aureus*, *Staphylococcus haemolyticus*, *Staphylococcus hominis* or *Staphylococcus lugdunensis*) because the difference values were < 0.28, whereas the culture result was *Staphylococcus aureus*. We additionally designed a short probe for specifically identifying *Staphylococcus aureus* (see “Methods” section). In this case, we used the short probe for *Staphylococcus aureus*, which only takes another 10 min, and identified that it was *Staphylococcus aureus*. In contrast, in patient No. 15, the Tm mapping result was genus *Staphylococcus*, whereas the culture result was coagulase-negative *Staphylococcus* (CNS), and the sequencing result was *Staphylococcus caprae*. We then used the short probe for *S. aureus* and found that it was not *S. aureus*. In addition, in patient No. 9, the culture results showed *Haemophilus influenzae*, *Klebsiella oxytoca*, and *Streptococcus pneumoniae,* while the Tm mapping and sequencing results showed only *Haemophilus influenzae.* This is because the Tm mapping method using IMLL Q-probes is able to identify only the dominant bacteria in a clinical sample.

**Table 3:** Individual results of identification starting from whole blood samples using a LightCycler 480 instrument (tube-to-tube variation ≤  ± 0.4 °C).

	Identification results				Identification level		Comments
	Tm mapping method using IMLL Q-probes	Diff.	Conventional culture method	Sequencing method*	Genus	Species	
Control: Healthy whole blood							
	None detected	**–**	No culture growth		**–**	**–**	Negative control
Patient							
1	*Bacillus cereus*	0.21	*Bacillus cereus*		**✔**	**✔**	
2	*Bacillus cereus*	0.45	*Bacillus cereus*		**✔**	**✔**	
3	*Escherichia coli*	0.30	*Escherichia coli*		**✔**	**✔**	
4	*Escherichia coli*	0.59	*Escherichia coli*		**✔**	**✔**	
5	*Escherichia coli*	0.36	*Escherichia coli* and *Klebsiella pneumoniae*	*Escherichia coli*	**✔**	**✔**	Dominant bacteria in polymicrobial infection
6	*Escherichia coli*	0.51	*Escherichia coli* and *Proteus mirabilis*	*Escherichia coli*	**✔**	**✔**	Dominant bacteria in polymicrobial infection
7	*Enterobacter aerogenes*	0.40	*Enterobacter aerogenes*		**✔**	**✔**	
8	*Enterococcus faecalis* or* Enterococcus faecium*	0.28 0.44	*Enterococcus faecalis*		**✔**		
9	*Haemophilus influenzae*	0.58	*Haemophilus influenzae* *Klebsiella oxytoca* *Streptococcus pneumoniae*	*Haemophilus influenzae*	**✔**	**✔**	Dominant bacteria in polymicrobial infection
10	*Klebsiella oxytoca*	0.40	*Klebsiella oxytoca*		**✔**	**✔**	
11	*Klebsiella pneumoniae*	0.33	*Klebsiella pneumoniae*		**✔**	**✔**	
12	*Pseudomonas aeruginosa*	0.52	*Pseudomonas aeruginosa*		**✔**	**✔**	
13	*Proteus mirabilis*	0.49	*Proteus mirabilis*		**✔**	**✔**	
14	*Serratia marcescens*	0.43	*Serratia marcescens*		**✔**	**✔**	
15	*genus Staphylococcus* (CNS)	0.38	coagulase negative *Staphylococcus* (CNS)	*Staphylococcus caprae*	**✔**		Short probe for *S. aureus* was additionally used
16	*genus Staphylococcus* (*S. aureus*)	0.28	*Staphylococcus aureus*		**✔**	(**✔**)	Short probe for *S. aureus* was additionally used
17	*genus Staphylococcus* (CNS)	0.41	coagulase negative *Staphylococcus* (CNS)	*Staphylococcus epidermidis*	**✔**		Short probe for *S. aureus* was additionally used
18	*genus Staphylococcus* (CNS)	0.55	*Staphylococcus epidermidis* and* Bacillus cereus*	*Staphylococcus epidermidis*	**✔**		Short probe for *S. aureus* was additionally used

Diff. , difference value. **✔**, Matched the culture/sequencing result. **–**, Did not perform a comparison with the culture/sequencing result. *Nucleotide sequence data are shown in the Supplementary Data File.

## Discussion

In the previous manuscript concerning the Tm mapping method, we mainly discussed the measurement error caused by the instrument, as the ranges of Tm values of the current Tm mapping method are < 4 °C^9^. In the current Tm mapping method, over 160 species of bacteria are registered in the database within a range of only 4 °C. Therefore, it is very significant to choose an analytical instrument for the Tm mapping method that can exactly measure Tm values with less measurement error. However, there are only a few optimal real-time PCR instruments for the current Tm mapping method, which requires a tube-to-tube variation of ≤  ± 0.1 °C, such as the RotorGeneQ or LightCycler® Nano.

To address this issue, we developed the improved Tm mapping method using IMLL Q-probes. The ranges of Tm values of the IMLL Q-probes exceed 20 °C, so real-time PCR instruments with a tube-to-tube variations of ≤  ± 0.5 °C are theoretically available for use with the improved Tm mapping method using IMLL Q-probes. The tube-to-tube variations of almost all commercially available real-time PCR instruments are from ± 0.3 to ± 0.5 °C, so the improved Tm mapping method using IMLL Q-probes can be performed with almost all real-time PCR instruments. As a result, the improved Tm mapping method using IMLL Q-probes is far superior to the previous Tm mapping method in terms of versatility of instrument, but the previous Tm mapping method is superior in identification accuracy and LOI (Limit of Identification). For example, the LOI results using *E. coli* were 1.25 vs. 5.0 CFU/PCR tube for the previous method vs. the improved method. However, since accuracy and reproducibility of these methods depend on the temperature control ability of the real-time PCR instrument, it is difficult to say which one is better.

Chakravorty et al. reported the rapid identification of bacterial isolates using a sloppy molecular beacon (SMB) melting temperature signature technique^11^. In their study, the authors used six Tm values as signatures of bacterial isolates to identify bacterial isolates using the D value as the distance between two points in six-dimensional space. The basic concept of the SMB melting temperature signature technique is similar to that of the Tm mapping method. In the present study, we referenced their SMB technique, a type of DNA probe. Tm variations in the SMB melting temperature signature technique depend on the number and position of probe-target mismatches on SMB hybridization. Therefore, the SMB melting temperature signature technique can be used to generate a wider variation range of Tm values than the current Tm mapping method, which means that tube-to-tube variation is not a major problem for the SMB melting temperature signature technique.

We then attempted to develop an improved Tm mapping method using long DNA probes. In this venture, in order to make a long probe (around 40-mer) without allowing the formation of secondary structures in the probes themselves, we first question whether or not molecular beacon structure was indispensable. To design an imperfect-match long probe, the target position in 16S ribosomal RNA gene is limited to some extent, but there is substantial flexibility in the design because the probe uses “imperfect-match” hybridization. By the way, degenerate probes are convenient for probe-target binding, but because multiple Tm values overlap, they are not useful for bacterial species identification using Tm values as in this method.

We designed and created many long probes using the delta G value and performed many operation checks until finally successfully choosing seven optimal long quenching probes without formation of secondary structure (i.e. without self-quenching). We named these long quenching probes the “imperfect-match linear long quenching probes (IMLL Q-probes)”. The major difference between the SMB melting temperature signature technique and the improved Tm mapping method using IMLL Q-probes is that the novel Tm mapping method performs nested PCR using bacterial universal primers and the eukaryote-made thermostable DNA polymerase and then uses IMLL Q-probes, thereby making it possible for PCR to identify bacterial isolates directly from patient samples within three hours of sample collection. Without the eukaryote-made thermostable DNA polymerase, it would be difficult to identify the bacterial isolate directly from whole blood samples or diagnose the absence of bacteria in a given sample. Namely, due to the superiority of eukaryote-made thermostable DNA polymerase and nested PCR method, the improved Tm mapping method has better LOI compared to the SMB melting temperature signature technique. As for versatility of instrument, both methods would be equally good. Regarding identification accuracy, it is difficult to say which one is better because it depends not only on the probe design but on the number and type of bacteria registered in the database. In addition, since accuracy and reproducibility of these methods depend on the temperature control ability of the real-time PCR instrument, it is also difficult to say which one is better.

Tm variations in the Tm mapping method using IMLL Q-probes depend on the number and position of probe-target mismatches on IMLL Q-probes hybridization. Therefore, the variation of Tm mapping shapes also depends on the number and position of probe-target mismatches on the hybridization. Table 1 shows the number of bacterial species registered in the database with a similar shape based on the difference value, demonstrating the identification accuracy of each species of bacteria based on mutual Tm mapping shape similarity. Each difference value was calculated using the mean of triplicate Tm values already measured by the Rotor-Gene Q instrument. This similarity does not always interfere with the Tm mapping identification, but a result of around 6 bacterial species (difference value ≤ 0.28) is difficult to narrow down to a single species using any optimal instruments (see Table 2). Even when using analytical instruments with tube-to-tube variations of ≤  ± 0.1 °C, bacterial species registered in the database that cannot be narrowed down to a single species included *Enterococcus faecalis* vs. *Enterococcus faecium* and *Staphylococcus aureus* vs. *Staphylococcus hemolyticus*, *Staphylococcus hominis*, and *Staphylococcus lugdunensis*. This is because there is a limit to the diversity of target base sequences of IMLL Q-probes when they belong to the same genus. However, for the bacteria mentioned above, most commercially available analytical instruments can narrow them down to at least the genus level. Concerning genus *Staphylococcus*, we made a short probe for specifically identifying *Staphylococcus aureus* and successfully distinguished *Staphylococcus aureus* from CNS (see Table 3), which only took another 10 min to perform.

In conclusion, the Tm mapping method using IMLL Q-probes enables the identification of the dominant bacteria in a clinical sample within three hours of whole blood collection. Notably, almost all commercially available real-time PCR instruments can be used with the improved Tm mapping method using the IMLL Q-probes, in contrast to the current Tm mapping method. Some bacterial species cannot be narrowed down to a single species, but they can at least be narrowed down to the genus level. In such cases, tailored short probes for identifying specific bacteria can be used, which only takes another 10 min to perform. The Tm mapping method using IMLL Q-probes is particularly useful for detecting infectious diseases, such as sepsis, that require prompt treatment, and is expected to contribute to the treatment of patients with severe infections.

## Methods

The Tm mapping method using IMLL Q-probes described here is an improvement of the Tm mapping method we previously reported^9^. Therefore, it should be noted that most of the methods are the same as the previously reported Tm mapping method.

### Clinical specimens

A total of 18 whole blood samples were collected from patients with sepsis at Toyama University Hospital and Nagaresugi Geriatric Hospital. In addition, a whole blood sample was collected from a single healthy volunteer at Toyama University Hospital. All procedures were performed under a protocol approved by the Ethics Committee at the University of Toyama and Nagaresugi Geriatric Hospital, and written informed consent was obtained from all patients. The methods were carried out in accordance with the approved guidelines.

### Isolation of bacterial genomic DNA from whole blood

A total of 2 mL of venous blood or, as a negative control for DNA extraction, 2 mL of molecular-grade distilled water (water deionized and sterilized for molecular biology; Nacalai Tesque, Inc., Kyoto, Japan) was collected in EDTA-2K tubes (BD Biosciences Japan, Tokyo, Japan). The blood samples were then centrifuged at 100 × *g* for 5 min to spin down the blood cells, and the resulting supernatant fractions (1 mL) were used. The supernatants were centrifuged again at 20,000 × *g* for 10 min, and 950 μL of the supernatant fractions was carefully removed, taking to avoid disturbing the pellets. Next, 1 mL of molecular-grade distilled water (water deionized and sterilized for molecular biology; Nacalai Tesque, Inc.) was added to the pellets, and the mixture was gently turned upside down several times and subsequently centrifuged at 20,000 × *g* for 5 min. Finally, 1 mL of the supernatant fractions was again carefully removed unless the pellet was resuspended before using the DNA extraction kit. DNA was isolated from the pellets using a DNA extraction kit (QIAamp UCP Pathogen Mini Kit; Qiagen) in accordance with the supplier’s instructions. Finally, bacterial DNA was eluted with 100 μL of elution buffer.

### Isolation of bacterial genomic DNA from bacterial colonies

The bacterial colonies were selected with a sterile inoculating loop and suspended in 1 mL of molecular-grade distilled water (water deionized and sterilized for molecular biology; Nacalai Tesque, Inc.). The samples were subsequently centrifuged at 20,000 × *g* for 10 min, and 950 μL of the supernatant was carefully removed while taking care to avoid disturbing the pellets. DNA was isolated from the resulting pellets using a DNA extraction kit (QIAamp UCP Pathogen Mini Kit; Qiagen) in accordance with the supplier’s instructions. Finally, bacterial DNA was eluted with 100 μL of elution buffer.

### PCR assays

The Veriti™ Thermal Cycler (Applied Biosystems, USA) was used for the amplification, and LightCycler® Nano (Roche Applied Science, Germany) was used for the Tm value analysis of the IMLL Q-probes. When using the LightCycler® Nano, which has two independent thermal blocks, we recommend using the same thermal block for all seven PCR tubes for Tm mapping identification. All PCR assays were performed as single-tube assays (no multiplex PCR). We used 1.5-mL PCR-clean Eppendorf tubes that were RNase- and DNase-free (Eppendorf, Germany), 0.2-mL PCR tubes (Qiagen) for the first PCR and 0.1-mL Strip Tubes and Caps (Qiagen) for the second (nested) PCR. All oligonucleotide primers were designed using a multiple alignment software program (ClustalX; Science Foundation Ireland, Dublin, Ireland) and were synthesized by Life Technologies Japan, Ltd. (Tokyo, Japan). All Q-probes were designed using a multiple alignment software program (ClustalX) and were synthesized by NIPPON STEEL & SUMIKIN Eco-Tech Corporation (Tsukuba, Japan). Bacterial universal primers were designed to universally amplify the seven regions of the bacterial 16S ribosomal RNA gene (Fig. 1b).

The primers were as follows: The first PCR primers (forward: 5′-AGAGTTTGATCATGGCTCAG-3′, reverse: 5′-CCGGGAACGTATTCACC-3′, amplicon size: 1378 bp), Region 1 primers (forward: 5′-AGAGTTTGATCATGGCTCAG-3′, reverse: 5′-CGTAGGAGTCTGGACCGT-3′, amplicon size: 338 bp), Region 2 primers (forward: 5′-GACTCCTACGGGAGGCA-3′, reverse: 5′-TATTACCGCGGCTGCTG-3′, amplicon size: 199 bp), Region 3 primers (forward: 5′-AGCAGCCGCGGTAATA-3′, reverse: 5′-GGACTACCAGGGTATCTAATCCT-3′, amplicon size: 287 bp), Region 4 primers (forward: 5′-AACAGGATTAGATACCCTGGTAG-3′, reverse: 5′-AATTAAACCACATGCTCCACC-3′, amplicon size: 181 bp), Region 5 primers (forward: 5′-TGGTTTAATTCGATGCAACGC-3′, reverse: 5′-GAGCTGACGACAGCCAT-3′, amplicon size: 120 bp). The Q-probes were as follows: IMLL Q-probe 1-1 (5′- GCCATCGGATGTGCCCAGATAAGATTAGCTAGTAGGTG-3′, probe size: 38 bp, delta G value: − 0.47 kcal/mol, binding site: 193–230 in *E. coli* 16S rRNA, Accession No. AB548582), IMLL Q-probe 1-2 (5′-AGGTAACGGCTTACTAAGGCAACGATCGTTAGCTGGTCTGAG-3′, probe size: 42 bp, delta G value: − 0.33 kcal/mol, binding site: 231–272 in *E. coli* 16S rRNA), IMLL Q-probe 2 (5′-GAATCTTCGACAATGGGGGAAAGCCTGATGGAGCCATGCCGCGTG-3′, probe size: 45 bp, delta G value: − 1.54 kcal/mol, binding site: 335–379 in *E. coli* 16S rRNA), IMLL Q-probe 3-1 (5′-GTAATACGGTGGGAGCTAGCGTTATTCGGAATTACAGGGCG-3′, probe size: 41 bp, delta G value: − 0.62 kcal/mol, binding site: 503–543 in *E. coli* 16S rRNA), IMLL Q-probe 3-2 (5′-GGCGGTTTGTTAAGTCAGTAGTGAAAGGCCCGGGCTCAACTTGG-3′, probe size: 44 bp, delta G value: − 0.63 kcal/mol, binding site: 557–600 in *E. coli* 16S rRNA), IMLL Q-probe 4 (5′-GTCCACGCTGTAAACGATGAGTATTAAGAGGTTGTGCC-3′, probe size: 38 bp, delta G value: − 0.95 kcal/mol, binding site: 776–813 in *E. coli* 16S rRNA), IMLL Q-probe 5 (5′-GAACCTTACCTAATCTTGACATCCTTAGAACTTTGCAGAGAT-3′, probe size: 42 bp, delta G value: − 0.99 kcal/mol, binding site: 949–990 in *E. coli* 16S rRNA), Q-probe for *S. aureus* (5′-GATCCGCGCTGCATTAGATA-3′, probe size: 20 bp, binding site: 211–230 in *S. aureus* 16S rRNA, Accession No. AB681291).

During the first PCR procedure, the PCR reaction mixture (20 µL) contained 2 µL of DNA template (DNA concentration varies depending on patient sample) in 200 µM of each dNTP (CleanAmp™ Hot Start dNTP Mix; Sigma-Aldrich, USA) filtered using an Amicon Ultra 50K centrifugal filter (Merck Millipore, Germany), 50 mM KCl, 2.25 mM MgCl_2_, 10 mM Tris–HCl (pH 8.3), 0.3 µM of each primer, and 1.0 units (0.5 µL) of eukaryote-made thermostable DNA polymerase supplemented with stock buffer solution. The generation of eukaryote-made thermostable DNA polymerase which is a recombinant using eukaryotic host cells (*Saccharomyces cerevisiae*) has been described in detail previously^10^. Although this eukaryote-made thermostable DNA polymerase may contain trace amounts of DNA from eukaryotic host cells as a result of its incomplete purification, it contains no bacterial DNA at all. In place of 2 µL of DNA template, the PCR reaction mixture contained 2 µL (8.0 ng/µL) of DNA extracted from *Escherichia coli* (ATCC 25922) as a positive control or 2 µL of molecular-grade distilled water (water deionized and sterilized for molecular biology; Nacalai Tesque, Inc.) as a negative control for the PCR step.

Each sample was incubated for five minutes at 95 °C to activate the Hot Start dNTPs and then denatured for 10 s at 94 °C, annealed for 10 s at 57 °C, and extended for 30 s at 72 °C for 40 cycles. The PCR product was diluted 100-fold with molecular-grade distilled water (water deionized and sterilized for molecular biology; Nacalai Tesque, Inc.) and then used as a template for the second (nested) PCR procedure.

For the second (nested) PCR procedure, the PCR reaction mixture (20 µL) contained 2 µL of DNA template of the diluted first PCR product in 200 µM of each dNTP (CleanAmp™ Hot Start dNTP Mix; Sigma-Aldrich) filtered using an Amicon Ultra 50K centrifugal filter (Merck Millipore), 50 mM KCl, 2.5 mM MgCl_2_, 10 mM Tris–HCl (pH 8.3), 0.75 µM of each forward primer, 0.25 µM of each reverse primer, and 1.0 units (0.5 µL) of eukaryote-made thermostable DNA polymerase supplemented with stock buffer solution. The 7 samples used to amplify Regions 1–5 were incubated for 5 min at 95 °C to activate the Hot Start dNTPs and then denatured for 10 s at 94 °C, annealed for 10 s at 57 °C, and extended for 10 s at 72 °C for 30 cycles.

### Tm value analyses

A total of 8 µL each of the 20 µL of second PCR amplicons was mixed with 0.12 µM of IMLL Q-probes (Total 10 µL). For the Tm value analysis, the resulting 7 mixtures were heated at 95 °C for 5 min, decreasing at 4 °C/s, and then cooled at 40 °C for 1 min. Tm value analyses were performed from 40 to 80 °C, increasing at 0.1 °C/step. The data profile was subsequently analyzed using the LightCycler® Nano software program (Roche Applied Science, Germany), and the Tm values were identified.

### Analytical sensitivity tests

The limits of identification and detection were determined by serially diluting (log_2_-fold) cultures with known counts (CFU) of *E. coli* in phosphate-buffered saline (PBS) and subjecting the samples to Tm mapping identification using IMLL Q-probes. The limits of identification were determined to be the final log_2_ dilution of the template in which the Tm mapping result was correct, with the correct number of Tm values and a difference value of ≤ 0.5. The limit of detection (LOD) was determined to be the final log_2_ dilution of the template in which at least one of the seven Tm values was observed.

### Nucleotide sequence-based analysis of bacterial genomic DNA

Amplicons from the samples used in the first PCR procedure were purified (QIAquick PCR Purification Kit; Qiagen) and then Sanger sequenced (3500 Genetic Analyzer; Applied Biosystems) using the Region 1 forward primer and the Region 5 reverse primer. An online homology search was performed for strain identification using the BLAST nucleotide database tool of the DNA Data Bank of Japan (http://www.ddbj.nig.ac.jp/index-j.html).

### Culture-based biochemical identification of bacteria

The whole blood samples (one aerobic blood culture bottle and one anaerobic blood culture bottle) were collected simultaneously with the blood sample for Tm analysis from the same puncture site. The whole blood samples were then analyzed according to standard methods used by the Clinical Laboratory Center (certified ISO15189) at Toyama University Hospital. The blood culture procedures were performed using the BacT/ALERT 3D system (bioMerieux, Inc., Mercy-l’Etoile, France). Positive blood culture bottles were subcultured in the appropriate media and incubated aerobically or anaerobically until sufficient growth was present to proceed with testing (usually 18–24 h). The specific identification methods differed according to the organism, although they included the MicroScan WalkAway system (Siemens Healthcare Diagnostics, IL, USA), RapID ANA II (Thermo Fisher SCIENTICIC, UK), and various latex agglutination and biochemical spot tests.

### Supplementary Information

Supplementary Information 1.

Supplementary Information 2.

## Data Availability

The microbial nucleotide sequence datasets generated during and/or analysed during the current study are available in the DNA Data Bank of Japan (DDBJ), Accession Number to dataset: LC773689, LC773690, LC773691, LC773692, LC773693, LC773694, LC773695, LC773696, LC773697, LC773698, LC773699, and LC773700 (each of which is shown in the Supplementary Data files). DDBJ temporary links for the reviewers to access the data: https://ddbj.nig.ac.jp/submission/submissions/64aa8a243a01a5005e74c7e9/mail_confirmation?token=3633a95c03c38aaac798306bdd8069dcf6d376e9.
