# Supplementary material for: Melting temperature mapping method using imperfect-match linear long probes
Source: Sci Rep. 2024 May 14;14:11055. doi: 10.1038/s41598-024-60987-7 (PMC11094154; doi:10.1038/s41598-024-60987-7)
Supplement: Supplementary file 1 — Supplementary Information 1. [file 41598_2024_60987_MOESM1_ESM.docx]

***Supplementary Information***

**Melting Temperature Mapping Method Using Imperfect-match Linear Long Probes**

Shinya Ootsuki^1^†, Hideki Niimi^1*^†, Tomohiro Ueno^2^, Masashi Mori^3^, Hiroshi Minami^4^, and Isao Kitajima^5*^

1. Clinical Laboratory and Transfusion Medicine & Cell Therapy Center,

Toyama University Hospital

2. Laboratory for Clinical Investigation, Osaka University Hospital

3. Research Institute for Bioresources and Biotechnology,

Ishikawa Prefectural University

4. Life Science Center, Hokkaido Mitsui Chemicals, Inc.

5. Administrative office, University of Toyama

*Correspondence to:

Hideki Niimi, M.D. Ph.D. E-mail address: hiniimi@med.u-toyama.ac.jp

Isao Kitajima, M.D. Ph.D. E-mail address: kitajima@med.u-toyama.ac.jp

Clinical Laboratory and Transfusion Medicine & Cell Therapy Center, Toyama University Hospital

2630 Sugitani Toyama 930-0194 JAPAN

Phone: (+81)-76-434-7759, Fax: (+81)-76-434-7759

† Both authors contributed equally to this work.

**Supplemental Table S1 (a)**

Sequence homology between the IMLL Q-probes and bacterial target regions (mismatched bases are colored in red, and surplus bases are colored in blue)

**IMLL Q-Probe 1-1** (5′➞3′)

| IMLL Q-Probe 1-1 target sequence | GCCATCGGATGTGCCCAGATAAGATTAGCTAGTAGGTG |
| --- | --- |
| *Acinetobacter baumanii* | GCTAATAGATGAGCCTAAGTCGGATTAGCTAGTTGGTG |
| *Acinetobacter calcoaceticus* | GCTAATAGATGAGCCTAAGTCGGATTAGCTAGTTGGTG |
| *Actinomyces israelii* | GCCGCATGGTGTGGCTGGGAAAGATTCACTTTTGTGGTG |
| *Aeromonas hydrophila* | GCGATTGGATATGCCCAGGTGGGATTAGCTAGTTGGTG |
| *Arthrobacter cumminsii* | GTTATCCGGAATTATTGGGCGTAAAGAGCTCGTAGGCG |
| *Bacillus cereus* | ACTTATGGATGGACCCGCGTCGCATTAGCTAGTTGGTG |
| *Bacteroides fragilis* | GGTAAAGGATGGGGATGCGTTCCATTAGGTTGTTGGTG |
| *Bacteroides nordii* | GGTAAAAGATGGGGATGCGTTCCATTAGGCAGTTGGCG |
| *Bacteroides thetaiotaomicron* | GTTATCGGATGGGGATGCGTTCCATTAGGCAGTTGGTG |
| *Bifidobacterium bifidum* | GGCGTGGGATGGGGTCGCGTCCTATCAGCTTGTTGGTG |
| *Bilophila wadsworthia* | GCTTAAGGATGAGTCCGCGTCCCATTAGCTAGTTGGCG |
| *Chryseobacterium gleum* | GGATAGAGATGGGCACGCGCAAGATTAGATAGTTGGTG |
| *Citrobacter amalonaticus* | GCCATCGGATGTGCCCAGATGGGATTAGCTAGTTGGTG |
| *Citrobacter freundii* | GCCATCGGATGTGCCCAGATGGGATTAGCTAGTAGGTG |
| *Clostridium difficile* | AGTACAGGATGGACCCGCGTCTGATTAGCTAGTTGGTA |
| *Clostridium leptum* | GCTCTGAGATGAGCTCGCGTCTGATTAGCTAGTTGGTC |
| *Corynebacterium striatum* | GGTGCAAGATGAGCTCGCGGCCTATCAGCTTGTTGGTG |
| *Eggerthella lenta* | GGCAAGGGATGGGGTCGCGGCCCATTAGGTAGTAGGCG |
| *Eikenella corrodens* | GTTATTCGAGCGGCCGATAACTGATTAGCTAGTTGGTG |
| *Enterobacter aerogenes* | GCCATCAGATGTGCCCAGATGGGATTAGCTAGTAGGTG |
| *Enterococcus avium* | ACTGATGGATGGACCCGCGGTGCATTAGCTAGTTGGTG |
| *Enterococcus casseliflavus* | ACTGATGGATGGACCCGCGGTGCATTAGCTAGTTGGTG |
| *Enterococcus faecalis* | GCTGATGGATGGACCCGCGGTGCATTAGCTAGTTGGTG |
| *Enterococcus faecium* | GCTGATGGATGGACCCGCGGTGCATTAGCTAGTTGGTG |
| *Escherichia albertii* | GCCATCGGATGTGCCCAGATGGGATTAGCTTGTTGGTG |
| *Escherichia coli* | GCCATCGGATGTGCCCAGATGGGATTAGCTAGTAGGTG |
| *Finegoldia magna* | GTCAT-AGATGGGCTCGCGTCTGATTAGCTAGTTGGTG |
| *Fusobacterium necrophorum* | GCTAAGAGAGAGCTTTGCGTCCCATTAGCTAGTTGGTG |
| *Gardnerella vaginalis* | GGCATGGGATGGGGTCGCGTCCTATCAGCTTGTAGCTG |
| *Gemella morbillorum* | ACTATGAGATGGCTTTGCGGTGCATTAGCTAGTTGGTG |
| *Haemophilus influenzae* | GCCATAGGATGAGCCCAAGTGGGATTAGGTAGTTGGTG |
| *Halomonas venusta* | GCTATTGGATGAGCCTATGTCGGATTAGCTAGTTGGTG |
| *Klebsiella oxytoca* | GCCATCGGATGTGCCCAGATGGGATTAGCTTGTAGGTG |
| *Klebsiella pneumoniae* | GCCATCAGATGTGCCCAGATGGGATTAGCTAGTAGGTG |
| *Lactobacillus crispatus* | GCTATGGGATGGCCCCGCGGTGCATTAGCTAGTTGGTA |
| *Lactobacillus jensenii* | GCTAAAGGATGGACCTGCGATGCATTAGCTAGTTGGTA |
| *Lactococcus garvieae* | ACTACTTGATGATCCCGCGTTGTATTAGCTAGTTGGTA |
| *Listeria monocytogenes* | GCTTACAGATGGGCCCGCGGTGCATTAGCTAGTTGGTA |
| *Morganella morganii* | GCCATCAGATGAACCCATATGGGATTAGCTAGTAGGTG |
| *Mycoplasma hominis* | ACTAAAAGATGAGGGTGCGGAACATTAGTTAGTTGGTG |
| *Nocardia cyriacigeorgica* | GGTGCGAGATGGGCCCGCGGCCTATCAGCTTGTTGGTG |
| *Odoribacter splanchnicus* | GGTATCGGATGGGCATGCGTCCTATTAGTTAGTTGGCG |
| *Parvimonas micra* | GGTGTAAGAAGGGCTCGCGTCTGATTAGCTAGTTGGAA |
| *Pasteurella multocida* | GCCATAAGATGAGCCCAAGTGGGATTAGGTAGTTGGTG |
| *Prevotella corporis* | GGTATGGGATGGGGATGCGTCTGATTAGCTTGTTGGCG |
| *Prevotella intermedia* | GGTGGAGGATGGGGATGCGTCTGATTAGCTTGTTGGTG |
| *Propionibacterium acnes* | GCTTTCGCCTGTGACGAAGCGTGAGTGACGGTAATGGG |
| *Proteus mirabilis* | ACTATCGGATGAACCCATATGGGATTAGCTAGTAGGTG |
| *Pseudomonas aeruginosa* | GCTATCAGATGAGCCTAGGTCGGATTAGCTAGTTGGTG |
| *Raoultella planticola* | GCCATCAGATGTGCCCAGATGGGATTAGCTAGTAGGTG |
| *Salmonella enterica* | GCCATCAGATGTGCCCAGATGGGATTAGCTTGTTGGTG |
| *Serratia marcescens* | GCCATCAGATGTGCCCAGATGGGATTAGCTAGTAGGTG |
| *Sphingomonas paucimobilis* | GCCTGAGGATGAGCCCGCGTTGGATTAGGTAGTTGGTG |
| *Staphylococcus aureus* | ACTTATAGATGGATCCGCGCTGCATTAGCTAGTTGGTA |
| *Staphylococcus capitis/epidermidis* | ACTTATAGATGGATCCGCGCCGCATTAGCTAGTTGGTA |
| *Staphylococcus cohnii* | ACTTATAGATGGACCCGCGCCGTATTAGCTAGTTGGTA |
| *Staphylococcus haemolyticus* | ACTTATAGATGGACCCGCGCCGTATTAGCTAGTTGGTA |
| *Staphylococcus hominis* | ACTTATAGATGGACCTGCGCCGTATTAGCTAGTTGGTA |
| *Staphylococcus lugdunensis* | ACTTATAGATGGACCCGCGCCGTATTAGCTAGTTGGTG |
| *Stenotrophomonas maltophilia* | GCGATTGAATGAGCCGATGTCGGATTAGCTAGTTGGCG |
| *Streptococcus agalactiae* | ACTGTGAGATGGACCTGCGTTGTATTAGCTAGTTGGTG |
| *Streptococcus anginosus* | GCTAGTAGATGGACCTGCGTTGTATTAGCTAGTAGGTA |
| *Streptococcus constellatus* | ACTACCAGATGGACCTGCGTTGTATTAGCTAGTTGGTG |
| *Streptococcus dysgalactiae* | ACTATGAGATGGACCTGCGTTGTATTAGCTAGTTGGTG |
| *Streptococcus gordonii* | ACTACCAGATGGACCTGCGTTGTATTAGCTAGTAGGTG |
| *Streptococcus mitis* | ACTACCAGATGGACCTGCGTTGTATTAGCTAGTTGGTG |
| *Streptococcus pneumoniae* | ACTACCAGATGGACCTGCGTTGTATTAGCTAGTTGGTG |
| *Streptococcus pyogenes* | ACTATGAGATGGACCTGCGTTGTATTAGCTAGTTGGTG |
| *Streptococcus salivarius* | ACTACAAGATGGACCTGCGTTGTATTAGCTAGTAGGTG |

**Supplemental Table S1 (b)**

**IMLL Q-Probe 1-2** (5′➞3′)

| IMLL Q-Probe 1-2 target sequence | AGGTAACGGCTTACTAAGGCAACGATCGTTAGCTGGTCTGAG |
| --- | --- |
| *Acinetobacter baumanii* | GGGTAAAGGCCTACCAAGGCGACGATCTGTAGCGGGTCTGAG |
| *Acinetobacter calcoaceticus* | GGGTAAAGGCCTACCAAGGCGACGATCTGTAGCGGGTCTGAG |
| *Actinomyces israelii* | GGGTGATGGCCTGCCAAGGCTTTGACGGGTAGCCGGCCTGAG |
| *Aeromonas hydrophila* | AGGTAATGGCTCACCAAGGCGACGATCCCTAGCTGGTCTGAG |
| *Arthrobacter cumminsii* | GGGTAATGGCCTACCAAGACGACGACGGGTAGCCGGCCTGAG |
| *Bacillus cereus* | AGGTAACGGCTCACCAAGGCAACGATGCGTAGCCGACCTGAG |
| *Bacteroides fragilis* | AGGTAACGGCTCACCAAGCCTTCGATGGATAGGGGTTCTGAG |
| *Bacteroides nordii* | GGGTAACGGCCCACCAAACCTTCGATGGATAGGGGTTCTGAG |
| *Bacteroides thetaiotaomicron* | AGGTAACGGCTCACCAAACCTTCGATGGATAGGGGTTCTGAG |
| *Bifidobacterium bifidum* | AGGTAACGGCTCACCAAGGCTTCGACGGGTAGCCGGCCTGAG |
| *Bilophila wadsworthia* | GGGTAACGGCCCACCAAGGCAACGATGGGTAGCCGGTCTGAG |
| *Chryseobacterium gleum* | AGGTAACGGCTCACCAAGTCTACGATCTTTAGGGGGCCTGAG |
| *Citrobacter amalonaticus* | AGGTAACGGCTCACCAAGGCGACGATCCCTAGCTGGTCTGAG |
| *Citrobacter freundii* | GGGTAACGGCTCACCTAGGCGACGATCCCTAGCTGGTCTGAG |
| *Clostridium difficile* | AGGTAACGGCTTACCAAGGCGACGATCAGTAGCCGACCTGAG |
| *Clostridium leptum* | GGGTAACGGCCCACCAAGGCGACGATCAGTAGCCGGACTGAG |
| *Corynebacterium striatum* | GGGTAATGGCCTACCAAGGCGTCGACGGGTAGCCGGCCTGAG |
| *Eggerthella lenta* | GGGTAACGGCCCACCTAGCCCGCGATGGGTAGCCGGGTTGAG |
| *Eikenella corrodens* | GGGTAAAGGCCTACCAAGGCGACGATCAGTAGCGGGTCTGAG |
| *Enterobacter aerogenes* | GGGTAATGGCTCACCTAGGCGACGATCCCTAGCTGGTCTGAG |
| *Enterococcus avium* | AGGTAACGGCTCACCAAGGCAACGATGCATAGCCGACCTGAG |
| *Enterococcus casseliflavus* | AGGTAACGGCTCACCAAGGCAACGATGCATAGCCGACCTGAG |
| *Enterococcus faecalis* | AGGTAACGGCTCACCAAGGCCACGATGCATAGCCGACCTGAG |
| *Enterococcus faecium* | AGGTAACGGCTCACCAAGGCCACGATGCATAGCCGACCTGAG |
| *Escherichia albertii* | AGGTAACGGCTCACCAAGGCGACGATCCCTAGCTGGTCTGAG |
| *Escherichia coli* | GGGTAACGGCTCACCTAGGCGACGATCCCTAGCTGGTCTGAG |
| *Finegoldia magna* | AGATAACAGCCCACCAAGGCGACGATCAGTAGCCGGTCTGAG |
| *Fusobacterium necrophorum* | AGGTAACGGCCCACCAAGGCAATGATGGGTAGCCGGCCTGAG |
| *Gardnerella vaginalis* | GGGTAATGGCCCACCTAGGCTTCGACGGGTAGCCGGCCTGAG |
| *Gemella morbillorum* | GGGTAAAGGCCCACCAAGGCGACGATGCATAGCCGACCTGAG |
| *Haemophilus influenzae* | GGGTAAAGGCCTACCAAGCCTGCGATCTCTAGCTGGTCTGAG |
| *Halomonas venusta* | AGGTAAAGGCTCACCAAGGCGACGATCCGTAGCTGGTCTGAG |
| *Klebsiella oxytoca* | AGGTAACGGCTCACCTAGGCGACGATCCCTAGCTGGTCTGAG |
| *Klebsiella pneumoniae* | GGGTAACGGCTCACCTAGGCGACGATCCCTAGCTGGTCTGAG |
| *Lactobacillus crispatus* | AGGTAAAGGCTTACCAAGGCGATGATGCATAGCCGAGTTGAG |
| *Lactobacillus jensenii* | AGGTAACGGCTTACCAAGGCGATGATGCATAGCCGAGTTGAG |
| *Lactococcus garvieae* | GTGTAAAGGACTACCAAGGCGATGATACATAGCCGACCTGAG |
| *Listeria monocytogenes* | GGGTAATGGCCTACCAAGGCAACGATGCATAGCCGACCTGAG |
| *Morganella morganii* | AGGTAACGGCTTACCTAGGCGACGATCCCTAGCTGGTCTGAG |
| *Mycoplasma hominis* | AGGTAATGGCCCACCAAGACTATGATGTTTAGCCGGGTCGAG |
| *Nocardia cyriacigeorgica* | GGGTAATGGCCTACCAAGGCGACGACGGGTAGCCGGCCTGAG |
| *Odoribacter splanchnicus* | GGGTAACAGCCCACCAAGACGATGATAGGTAGGGGTTCTGAG |
| *Parvimonas micra* | GGGTAAAGGCCTACCAAGGCGACGATCAGTAGCCGGTCTGAG |
| *Pasteurella multocida* | GGGTAAAGGCCTACCAAGCCTGCGATCTCTAGCTGGTCTGAG |
| *Prevotella corporis* | GGGTAACGGCCCACCAAGGCATCGATCAGTAGGGGTTCTGAG |
| *Prevotella intermedia* | GGGTAACGGCCCACCAAGGCTACGATCAGTAGGGGTTCTGAG |
| *Propionibacterium acnes* | GGGTAGTGGCTTACCAAGGCTTTGACGGGTAGCCGGCCTGAG |
| *Proteus mirabilis* | GGGTAAAGGCTCACCTAGGCGACGATCTCTAGCTGGTCTGAG |
| *Pseudomonas aeruginosa* | GGGTAAAGGCCTACCAAGGCGACGATCCGTAACTGGTCTGAG |
| *Raoultella planticola* | GGGTAATGGCTCACCTAGGCGACGATCCCTAGCTGGTCTGAG |
| *Salmonella enterica* | AGGTAACGGCTCACCAAGGCGACGATCCCTAGCTGGTCTGAG |
| *Serratia marcescens* | GGGTAATGGCTCACCTAGGCGACGATCCCTAGCTGGTCTGAG |
| *Sphingomonas paucimobilis* | AGGTAACGGCTTACCAAGGCAACGATGCGTAGCCGACCTGAG |
| *Staphylococcus aureus* | AGGTAACGGCTTACCAAGGCAACGATGCATAGCCGACCTGAG |
| *Staphylococcus capitis/epidermidis* | AGGTAACGGCTTACCAAGGCAACGATACGTAGCCGACCTGAG |
| *Staphylococcus cohnii* | AGGTAACGGCTTACCAAGGCAACGATGCGTAGCCGACCTGAG |
| *Staphylococcus haemolyticus* | AGGTAACGGCTTACCAAGGCGACGATACGTAGCCGACCTGAG |
| *Staphylococcus hominis* | AGGTAACGGCTTACCAAGGCAACGATACGTAGCCGACCTGAG |
| *Staphylococcus lugdunensis* | AGGTAACGGCTCACCAAGGCAACGATACGTAGCCGACCTGAG |
| *Stenotrophomonas maltophilia* | GGGTAAAGGCCCACCAAGGCGACGATCCGTAGCTGGTCTGAG |
| *Streptococcus agalactiae* | AGGTAAAGGCTCACCAAGGCGACGATACATAGCCGACCTGAG |
| *Streptococcus anginosus* | GGGTAATGGCCTACCTAGGCGACGATACATAGCCGACCTGAG |
| *Streptococcus constellatus* | AGGTAACGGCTCACCAAGGCAACGATACATAGCCGACCTGAG |
| *Streptococcus dysgalactiae* | AGGTAACGGCTCACCAAGGCGACGATACATAGCCGACCTGAG |
| *Streptococcus gordonii* | AGGTAACGGCTCACCTAGGCGACGATACATAGCCGACCTGAG |
| *Streptococcus mitis* | GGGTAACGGCTCACCAAGGCGACGATACATAGCCGACCTGAG |
| *Streptococcus pneumoniae* | GGGTAACGGCTCACCAAGGCGACGATACATAGCCGACCTGAG |
| *Streptococcus pyogenes* | AGGTAAAGGCTCACCAAGGCGACGATACATAGCCGACCTGAG |
| *Streptococcus salivarius* | AGGTAACGGCTCACCTAGGCGACGATACATAGCCGACCTGAG |

**Supplemental Table S1 (c)**

**IMLL Q-Probe 2** (5′➞3′)

| IMLL Q-Probe 2 target sequence | GAATCTTCGACAATGGGGGAAAGCCTGATGGAGCCATGCCGCGTG |
| --- | --- |
| *Acinetobacter baumanii* | GAATATTGGACAATGGGGGGAACCCTGATCCAGCCATGCCGCGTG |
| *Acinetobacter calcoaceticus* | GAATATTGGACAATGGGCGCAAGCCTGATCCAGCCATGCCGCGTG |
| *Actinomyces israelii* | GGGTATTGCACAATGGGCGCAAGCCTGATGCAGCGACGTCGCGTG |
| *Aeromonas hydrophila* | GAATATTGCACAATGGGGGAAACCCTGATGCAGCCATGCCGCGTG |
| *Arthrobacter cumminsii* | GAATATTGCACAATGGGCGCAAGCCTGATGCAGCGACGCCGCGTG |
| *Bacillus cereus* | GAATCTTCCGCAATGGACGAAAGTCTGACGGAGCAACGCCGCGTG |
| *Bacteroides fragilis* | GAATATTGGTCAATGGGCGCTAGCCTGAACCAGCCAAGTAGCGTG |
| *Bacteroides nordii* | GAATATTGGTCAATGGGCGAAAGCCTGAACCAGCCAAGTAGCGTG |
| *Bacteroides thetaiotaomicron* | GAATATTGGTCAATGGGCGCAGGCCTGAACCAGCCAAGTAGCGTG |
| *Bifidobacterium bifidum* | GAATATTGCACAATGGGCGCAAGCCTGATGCAGCGACGCCGCGTG |
| *Bilophila wadsworthia* | GAATATTGCGCAATGGGCGAAAGCCTGACGCAGCGACGCCGCGTG |
| *Chryseobacterium gleum* | GAATATTGGACAATGGGTGCGAGCCTGATCCAGCCATCCCGCGTG |
| *Citrobacter amalonaticus* | GAATATTGCACAATGGGCGCAAGCCTGATGCAGCCATGCCGCGTG |
| *Citrobacter freundii* | GAATATTGCACAATGGGCGCAAGCCTGATGCAGCCATGCCGCGTG |
| *Clostridium difficile* | GAATATTGCACAATGGGCGAAAGCCTGATGCAGCAACGCCGCGTG |
| *Clostridium leptum* | GAATATTGGGCAATGGGCGCAAGCCTGACCCAGCAACGCCGCGTG |
| *Corynebacterium striatum* | GAATATTGCACAATGGGCGGAAGCCTGATGCAGCGACGCCGCGTG |
| *Eggerthella lenta* | GAATTTTGCGCAATGGGGGAAACCCTGACGCAGCAACGCCGCGTG |
| *Eikenella corrodens* | GAATTTTGGACAATGGGGGCAACCCTGATCCAGCCATGCCGCGTG |
| *Enterobacter aerogenes* | GAATATTGCACAATGGGCGCAAGCCTGATGCAGCCATGCCGCGTG |
| *Enterococcus avium* | GAATCTTCGGCAATGGACGCAAGTCTGACCGAGCAACGCCGCGTG |
| *Enterococcus casseliflavus* | GAATCTTCGGCAATGGACGAAAGTCTGACCGAGCAACGCCGCGTG |
| *Enterococcus faecalis* | GAATCTTCGGCAATGGACGAAAGTCTGACCGAGCAACGCCGCGTG |
| *Enterococcus faecium* | GAATCTTCGGCAATGGACGAAAGTCTGACCGAGCAACGCCGCGTG |
| *Escherichia albertii* | GAATATTGCACAATGGGCGCAAGCCTGATGCAGCCATGCCGCGTG |
| *Escherichia coli* | GAATATTGCACAATGGGCGCAAGCCTGATGCAGCCATGCCGCGTG |
| *Finegoldia magna* | GAATATTGCACAATGGGGGAAACCCTGATGCAGCGACGCCGCGTG |
| *Fusobacterium necrophorum* | GAATATTGGACAATGGACCAAAGTCTGATCCAGCAATTCTGTGTG |
| *Gardnerella vaginalis* | GAATATTGCGCAATGGGGGAAACCCTGACGCAGCGACGCCGCGTG |
| *Gemella morbillorum* | GAATCTTCCGCAATGGGCGAAAGCCTGACGGAGCAACGCCGCGTG |
| *Haemophilus influenzae* | GAATATTGCGCAATGGGGGGAACCCTGACGCAGCCATGCCGCGTG |
| *Halomonas venusta* | GAATATTGGACAATGGGGGCAACCCTGATCCAGCCATGCCGCGTG |
| *Klebsiella oxytoca* | GAATATTGCACAATGGGCGCAAGCCTGATGCAGCCATGCCGCGTG |
| *Klebsiella pneumoniae* | GAATATTGCACAATGGGCGCAAGCCTGATGCAGCCATGCCGCGTG |
| *Lactobacillus crispatus* | GAATCTTCCACAATGGACGCAAGTCTGATGGAGCAACGCCGCGTG |
| *Lactobacillus jensenii* | GAATCTTCCACAATGGACGCAAGTCTGATGGAGCAACGCCGCGTG |
| *Lactococcus garvieae* | GAATCTTCGGCAATGGGGGCAACCCTGACCGAGCAACGCCGCGTG |
| *Listeria monocytogenes* | GAATCTTCCGCAATGGACGAAAGTCTGACGGAGCAACGCCGCGTG |
| *Morganella morganii* | GAATATTGCACAATGGGCGCAAGCCTGATGCAGCCATGCCGCGTG |
| *Mycoplasma hominis* | GAATATTCCACAATGAGCGAAAGCTTGATGGAGCGACACAGCGTG |
| *Nocardia cyriacigeorgica* | GAATATTGCACAATGGGCGAAAGCCTGATGCAGCGACGCCGCGTG |
| *Odoribacter splanchnicus* | GAATATTGGTCAATGGACGTAAGTCTGAACCAGCCAAGTCGCGTG |
| *Parvimonas micra* | GAATATTGCACAATGGGGGGAACCCTGATGCAGCGACGCCGCGTG |
| *Pasteurella multocida* | GAATATTGCGCAATGGGGGGAACCCTGACGCAGCCATGCCGCGTG |
| *Prevotella corporis* | GAATATTGGTCAATGGGCGCTAGCCTGAACCAGCCAAGTAGCGTG |
| *Prevotella intermedia* | GAATATTGGTCAATGGACGTAAGTCTGAACCAGCCAAGTAGCGTG |
| *Propionibacterium acnes* | GAATATTGCACAATGGGCGGAAGCCTGATGCAGCAACGCCGCGTG |
| *Proteus mirabilis* | GAATATTGCACAATGGGCGCAAGCCTGATGCAGCCATGCCGCGTG |
| *Pseudomonas aeruginosa* | GAATATTGGACAATGGGCGAAAGCCTGATCCAGCCATGCCGCGTG |
| *Raoultella planticola* | GAATATTGCACAATGGGCGCAAGCCTGATGCAGCCATGCCGCGTG |
| *Salmonella enterica* | GAATATTGCACAATGGGCGCAAGCCTGATGCAGCCATGCCGCGTG |
| *Serratia marcescens* | GAATATTGCACAATGGGCGCAAGCCTGATGCAGCCATGCCGCGTG |
| *Sphingomonas paucimobilis* | GAATATTGGACAATGGGCGAAAGCCTGATCCAGCAATGCCGCGTG |
| *Staphylococcus aureus* | GAATCTTCCGCAATGGGCGAAAGCCTGACGGAGCAACGCCGCGTG |
| *Staphylococcus capitis/epidermidis* | GAATCTTCCGCAATGGGCGAAAGCCTGACGGAGCAACGCCGCGTG |
| *Staphylococcus cohnii* | GAATCTTCCGCAATGGGCGAAAGCCTGACGGAGCAACGCCGCGTG |
| *Staphylococcus haemolyticus* | GAATCTTCCGCAATGGGCGAAAGCCTGACGGAGCAACGCCGCGTG |
| *Staphylococcus hominis* | GAATCTTCCGCAATGGGCGAAAGCCTGACGGAGCAACGCCGCGTG |
| *Staphylococcus lugdunensis* | GAATCTTCCGCAATGGGCGAAAGCCTGACGGAGCAACGCCGCGTG |
| *Stenotrophomonas maltophilia* | GAATATTGGACAATGGGCGCAAGCCTGATCCAGCCATACCGCGTG |
| *Streptococcus agalactiae* | GAATCTTCGGCAATGGACGGAAGTCTGACCGAGCAACGCCGCGTG |
| *Streptococcus anginosus* | GAATCTTCGGCAATGGGGGGAACCCTGACCGAGCAACGCCGCGTG |
| *Streptococcus constellatus* | GAATCTTCGGCAATGGGGGGAACCCTGACCGAGCAACGCCGCGTG |
| *Streptococcus dysgalactiae* | GAATCTTCGGCAATGGACGGAAGTCTGACCGAGCAACGCCGCGTG |
| *Streptococcus gordonii* | GAATCTTCGGCAATGGACGAAAGTCTGACCGAGCAACGCCGCGTG |
| *Streptococcus mitis* | GAATCTTCGGCAATGGACGGAAGTCTGACCGAGCAACGCCGCGTG |
| *Streptococcus pneumoniae* | GAATCTTCGGCAATGGACGGAAGTCTGACCGAGCAACGCCGCGTG |
| *Streptococcus pyogenes* | GAATCTTCGGCAATGGGGGCAACCCTGACCGAGCAACGCCGCGTG |
| *Streptococcus salivarius* | GAATCTTCGGCAATGGGGGCAACCCTGACCGAGCAACGCCGCGTG |

**Supplemental Table S1 (d)**

**IMLL Q-Probe 3-1** (5′➞3′)

| IMLL Q-Probe 3-1 target sequence | GTAATACGGTGGGAGCTAGCGTTATTCGGAATTACAGGGCG |
| --- | --- |
| *Acinetobacter baumanii* | GTAATACAGAGGGTGCGAGCGTTAATCGGATTTACTGGGCG |
| *Acinetobacter calcoaceticus* | GTAATACAGAGGGTGCAAGCGTTAATCGGATTTACTGGGCG |
| *Actinomyces israelii* | GTAATACGTAGGGCGCGAGCGTTGTCCGGAATTATTGGGCG |
| *Aeromonas hydrophila* | GTAATACGGAGGGTGCAAGCGTTAATCGGAATTACTGGGCG |
| *Arthrobacter cumminsii* | GTAATACGTAGGGCGCGAGCGTTATCCGGAATTATTGGGCG |
| *Bacillus cereus* | GTAATACGTAGGTGGCAAGCGTTATCCGGAATTATTGGGCG |
| *Bacteroides fragilis* | GTAATACGGAGGATCCGAGCGTTATCCGGATTTATTGGGTT |
| *Bacteroides nordii* | GTAATACGGAGGATCCGAGCGTTATCCGGATTTATTGGGTT |
| *Bacteroides thetaiotaomicron* | GTAATACGGAGGATCCGAGCGTTATCCGGATTTATTGGGTT |
| *Bifidobacterium bifidum* | GTAATACGTAGGGCGCAAGCGTTATCCGGATTTATTGGGCG |
| *Bilophila wadsworthia* | GTAATACGGAGGGTGCAAGCGTTAATCGGAATCACTGGGCG |
| *Chryseobacterium gleum* | GTAATACGGAGGGTGCAAGCGTTATCCGGATTTATTGGGTT |
| *Citrobacter amalonaticus* | GTAATACGGAGGGTGCAAGCGTTAATCGGAATTACTGGGCG |
| *Citrobacter freundii* | GTAATACGGAGGGTGCAAGCGTTAATCGGAATTACTGGGCG |
| *Clostridium difficile* | GTAATACGTAGGGGGCTAGCGTTATCCGGATTTACTGGGCG |
| *Clostridium leptum* | GTAATACGTAGGGGGCAAGCGTTATCCGGATTTACTGGGTG |
| *Corynebacterium striatum* | GTAATACGTAGGGTGCGAGCGTTGTCCGGAATTACTGGGCG |
| *Eggerthella lenta* | GTAATACGTAGGGAGCGAGCGTTATCCGGATTCATTGGGCG |
| *Eikenella corrodens* | GTAATACGTAGGGTGCGAGCGTTAATCGGAATTACTGGGCG |
| *Enterobacter aerogenes* | GTAATACGGAGGGTGCAAGCGTTAATCGGAATTACTGGGCG |
| *Enterococcus avium* | GTAATACGTAGGTGGCAAGCGTTGTCCGGATTTATTGGGCG |
| *Enterococcus casseliflavus* | GTAATACGTAGGTGGCAAGCGTTGTCCGGATTTATTGGGCG |
| *Enterococcus faecalis* | GTAATACGTAGGTGGCAAGCGTTGTCCGGATTTATTGGGCG |
| *Enterococcus faecium* | GTAATACGTAGGTGGCAAGCGTTGTCCGGATTTATTGGGCG |
| *Escherichia albertii* | GTAATACGGAGGGTGCAAGCGTTAATCGGAATTACTGGGCG |
| *Escherichia coli* | GTAATACGGAGGGTGCAAGCGTTAATCGGAATTACTGGGCG |
| *Finegoldia magna* | GTAATACGTATGGAGCGAGCGTTGTCCGGAATTATTGGGCG |
| *Fusobacterium necrophorum* | GTAATACGTATGTCGCAAGCGTTATCCGGATTTATTGGGCG |
| *Gardnerella vaginalis* | GTAATACGTAGGGCGCAAGCGTTATCCGGAATTATTGGGCG |
| *Gemella morbillorum* | GTAATACGTAGGTGGCAAGCGTTGTCCGGAATTATTGGGCG |
| *Haemophilus influenzae* | GTAATACGGAGGGTGCGAGCGTTAATCGGAATAACTGGGCG |
| *Halomonas venusta* | GTAATACGGAGGGTGCAAGCGTTAATCGGAATTACTGGGCG |
| *Klebsiella oxytoca* | GTAATACGGAGGGTGCAAGCGTTAATCGGAATTACTGGGCG |
| *Klebsiella pneumoniae* | GTAATACGGAGGGTGCAAGCGTTAATCGGAATTACTGGGCG |
| *Lactobacillus crispatus* | GTAATACGTAGGTGGCAAGCGTTGTCCGGATTTATTGGGCG |
| *Lactobacillus jensenii* | GTAATACGTAGGTGGCAAGCGTTGTCCGGATTTATTGGGCG |
| *Lactococcus garvieae* | GTAATACGTAGGTCCCAAGCGTTGTCCGGATTTATTGGGCG |
| *Listeria monocytogenes* | GTAATACGTAGGTGGCAAGCGTTGTCCGGATTTATTGGGCG |
| *Morganella morganii* | GTAATACGGAGGGTGCAAGCGTTAATCGGAATTACTGGGCG |
| *Mycoplasma hominis* | GTAATACATAGGTCGCAAGCGTTATCCGGAATTATTGGGCG |
| *Nocardia cyriacigeorgica* | GTAATACGTAGGGTGCGAGCGTTGTCCGGAATTACTGGGCG |
| *Odoribacter splanchnicus* | GTAATACGGAGGATGCGAGCGTTATCCGGATTTATTGGGTT |
| *Parvimonas micra* | GTAATACGTATGGGGCGAGCGTTGTCCGGAATTATTGGGCG |
| *Pasteurella multocida* | GTAATACGGAGGGTGCGAGCGTTAATCGGAATAACTGGGCG |
| *Prevotella corporis* | GTAATACGGAAGGTCCGGGCGTTATCCGGATTTATTGGGTT |
| *Prevotella intermedia* | GTAATACGGAAGGTCCAGGCGTTATCCGGATTTATTGGGTT |
| *Propionibacterium acnes* | GTGATACGTAGGGTGCGAGCGTTGTCCGGATTTATTGGGCG |
| *Proteus mirabilis* | GTAATACGGAGGGTGCAAGCGTTAATCGGAATTACTGGGCG |
| *Pseudomonas aeruginosa* | GTAATACGAAGGGTGCAAGCGTTAATCGGAATTACTGGGCG |
| *Raoultella planticola* | GTAATACGGAGGGTGCAAGCGTTAATCGGAATTACTGGGCG |
| *Salmonella enterica* | GTAATACGGAGGGTGCAAGCGTTAATCGGAATTACTGGGCG |
| *Serratia marcescens* | GTAATACGGAGGGTGCAAGCGTTAATCGGAATTACTGGGCG |
| *Sphingomonas paucimobilis* | GTAATACGGAGGGGGCTAGCGTTGTTCGGAATTACTGGGCG |
| *Staphylococcus aureus* | GTAATACGTAGGTGGCAAGCGTTATCCGGAATTATTGGGCG |
| *Staphylococcus capitis/epidermidis* | GTAATACGTAGGTGGCAAGCGTTATCCGGAATTATTGGGCG |
| *Staphylococcus cohnii* | GTAATACGTAGGTGGCAAGCGTTATCCGGAATTATTGGGCG |
| *Staphylococcus haemolyticus* | GTAATACGTAGGTGGCAAGCGTTATCCGGAATTATTGGGCG |
| *Staphylococcus hominis* | GTAATACGTAGGTGGCAAGCGTTATCCGGAATTATTGGGCG |
| *Staphylococcus lugdunensis* | GTAATACGTAGGTGGCAAGCGTTATCCGGAATTATTGGGCG |
| *Stenotrophomonas maltophilia* | GTAATACGAAGGGTGCAAGCGTTACTCGGAATTACTGGGCG |
| *Streptococcus agalactiae* | GTAATACGTAGGTCCCGAGCGTTGTCCGGATTTATTGGGCG |
| *Streptococcus anginosus* | GTAATACGTAGGTCCCGAGCGTTGTCCGGATTTATTGGGCG |
| *Streptococcus constellatus* | GTAATACGTAGGTCCCGAGCGTTGTCCGGATTTATTGGGCG |
| *Streptococcus dysgalactiae* | GTAATACGTAGGTCCCGAGCGTTGTCCGGATTTATTGGGCG |
| *Streptococcus gordonii* | GTAATACGTAGGTCCCGAGCGTTGTCCGGATTTATTGGGCG |
| *Streptococcus mitis* | GTAATACGTAGGTCCCGAGCGTTGTCCGGATTTATTGGGCG |
| *Streptococcus pneumoniae* | GTAATACGTAGGTCCCGAGCGTTGTCCGGATTTATTGGGCG |
| *Streptococcus pyogenes* | GTAATACGTAGGTCCCGAGCGTTGTCCGGATTTATTGGGCG |
| *Streptococcus salivarius* | GTAATACGTAGGTCCCGAGCGTTGTCCGGATTTATTGGGCG |

**Supplemental Table S1 (e)**

**IMLL Q-Probe 3-2** (5′➞3′)

| IMLL Q-Probe 3-2 target sequence | GGCGGTTTGTTAAGTCAGTAGTGAAAGGCCCGGGCTCAACTTGG |
| --- | --- |
| *Acinetobacter baumanii* | GGCGGCTTATTAAGTCGGATGTGAAATCCCCGAGCTTAACTTGG |
| *Acinetobacter calcoaceticus* | GGCGGCTAATTAAGTCAAATGTGAAATCCCCGAGCTTAACTTGG |
| *Actinomyces israelii* | GGCGGCTGGTCGCGTCTGCCGTGAAATCCTCTGGCTCAGCTGGG |
| *Aeromonas hydrophila* | GGCGGTTGGATAAGTTAGATGTGAAAGCCCCGGGCTCAACCTGG |
| *Arthrobacter cumminsii* | GGCGGTTTGTCGCGTCTGCTGTGAAAGCCCGGGGCTTAACTCCG |
| *Bacillus cereus* | GGTGGTTTCTTAAGTCTGATGTGAAAGCCCACGGCTCAACCGTG |
| *Bacteroides fragilis* | GGTGGACTGGTAAGTCAGTTGTGAAAGTTTGCGGCTCAACCGTA |
| *Bacteroides nordii* | GGTGGACATGTAAGTCAGTTGTGAAAGTTTGCGGCTCAACCGTA |
| *Bacteroides thetaiotaomicron* | GGTGGACAGTTAAGTCAGTTGTGAAAGTTTGCGGCTCAACCGTA |
| *Bifidobacterium bifidum* | GGCGGCTCGTCGCGTCCGGTGTGAAAGTCCATCGCTTAACGGTG |
| *Bilophila wadsworthia* | GGCGGCTTGGTAAGTCAGGGGTGAAATCCCACAGCCCAACTGTG |
| *Chryseobacterium gleum* | GGCGGATGTGTAAGTCAGTGGTGAAATCTCACAGCTTAACTGTG |
| *Citrobacter amalonaticus* | GGCGGTCTGTCAAGTCGGATGTGAAATCCCCGGGCTCAACCTGG |
| *Citrobacter freundii* | GGCGGTCTGTCAAGTCGGATGTGAAATCCCCGGGCTCAACCTGG |
| *Clostridium difficile* | GGCGGTCTTTCAAGTCAGGAGTGAAAGGCTACGGCTCAACCGTA |
| *Clostridium leptum* | GGCGGCGAGGCAAGTCAGGCGTGAAATCTATGGGCTTAACCCAT |
| *Corynebacterium striatum* | GGTGGTTTGTCGCGTCGTCTGTGAAATTCCGGGGCTTAACTCCG |
| *Eggerthella lenta* | GGCGGCCTCTCAAGCGGGATCTCTAATCCGAGGGCTCAACCCCC |
| *Eikenella corrodens* | GACGGTTATTTAAGCAGGATGTGAAATCCCCGGGCTTAACCTGG |
| *Enterobacter aerogenes* | GGCGGTCTGTCAAGTCGGATGTGAAATCCCCGGGCTCAACCTGG |
| *Enterococcus avium* | GGCGGTTTCTTAAGTCTGATGTGAAAGCCCCCGGCTCAACCGGG |
| *Enterococcus casseliflavus* | GGCGGTTTCTTAAGTCTGATGTGAAAGCCCCCGGCTCAACCGGG |
| *Enterococcus faecalis* | GGCGGTTTCTTAAGTCTGATGTGAAAGCCCCCGGCTCAACCGGG |
| *Enterococcus faecium* | GGCGGTTTCTTAAGTCTGATGTGAAAGCCCCCGGCTCAACCGGG |
| *Escherichia albertii* | GGCGGTTGATTAAGTCAGATGTGAAATCCCCGGGCTCAACCTGG |
| *Escherichia coli* | GGCGGTTTGTTAAGTCAGATGTGAAATCCCCGGGCTCAACCTGG |
| *Finegoldia magna* | GGCGGTTTAATAAGTCGAATGTTAAAGATCGGGGCTCAACCCCG |
| *Fusobacterium necrophorum* | GGCGGCAAGGAAAGTCTGATGTGAAAATGCGGAGCTCAACTCCG |
| *Gardnerella vaginalis* | GGCGGTTCGTCGCGTCTGGTGTGAAAGCCCATCGCTTAACGGTG |
| *Gemella morbillorum* | GGTGGTTTAATAAGTCTGATGTGAAAGCCCACGGCTCAACCGTG |
| *Haemophilus influenzae* | GGCGGTTATTTAAGTGAGGTGTGAAAGCCCCGGGCTTAACCTGG |
| *Halomonas venusta* | GGTGGCTTGATAAGCCGGTTGTGAAAGCCCCGGGCTCAACCTGG |
| *Klebsiella oxytoca* | GGCGGTCTGTCAAGTCGGATGTGAAATCCCCGGGCTCAACCTGG |
| *Klebsiella pneumoniae* | GGCGGTCTGTCAAGTCGGATGTGAAATCCCCGGGCTCAACCTGG |
| *Lactobacillus crispatus* | GGCGGAAGAATAAGTCTGATGTGAAAGCCCTCGGCTTAACCGAG |
| *Lactobacillus jensenii* | GGCGGATTGATAAGTCTGATGTGAAAGCCTTCGGCTCAACCGAA |
| *Lactococcus garvieae* | GGTGGTTTCTTAAGTCTGATGTAAAAGGCAGTGGCTCAACCATT |
| *Listeria monocytogenes* | GGCGGTCTTTTAAGTCTGATGTGAAAGCCCCCGGCTTAACCGGG |
| *Morganella morganii* | GGCGGTTGATTGAGTCAGATGTGAAATCCCCGGGCTTAACCCGG |
| *Mycoplasma hominis* | GGCTGTTTGTTAAGTCTGGAGTTAAATCCCGGGGCTCAACCCCG |
| *Nocardia cyriacigeorgica* | GGCGGCTTGTCGCGTCGATCGTGAAAACTTGGGGCTCAACCCCA |
| *Odoribacter splanchnicus* | GGCGGTTTATTAAGTTAGTGGTTAAATATTTGAGCTCAACTCAA |
| *Parvimonas micra* | GGCGGTTTTTTAAGTCAGGTGTGAAAGCGTGAGGCTTAACCTCA |
| *Pasteurella multocida* | GGCGGACTTTTAAGTGAGATGTGAAATCCCCGAGCTTAACTTGG |
| *Prevotella corporis* | GGCGGCCTGTTAAGCGTGTTGTGAAATGTAGATGCTCAACATCT |
| *Prevotella intermedia* | GGCGGTCTGTTAAGCGTGTTGTGAAATTTAGGTGCTCAACATCT |
| *Propionibacterium acnes* | GGTGGTTGATCGCGTCGGAAGTGTAATCTTGGGGCTTAACCCTG |
| *Proteus mirabilis* | GGCGGTCAATTAAGTCAGATGTGAAAGCCCCGAGCTTAACTTGG |
| *Pseudomonas aeruginosa* | GGTGGTTCAGCAAGTTGGATGTGAAATCCCCGGGCTCAACCTGG |
| *Raoultella planticola* | GGCGGTTTGTTAAGTCAGATGTGAAATCCCCGGGCTCAACCTGG |
| *Salmonella enterica* | GGCGGTCTGTCAAGTCGGATGTGAAATCCCCGGGCTCAACCTGG |
| *Serratia marcescens* | GGCGGTTTGTTAAGTCAGATGTGAAATCCCCGGGCTCAACCTGG |
| *Sphingomonas paucimobilis* | GGCGGCTTTGTAAGTCAGAGGTGAAAGCCTGGAGCTCAACTCCA |
| *Staphylococcus aureus* | GGCGGTTTTTTAAGTCTGATGTGAAAGCCCACGGCTCAACCGTG |
| *Staphylococcus capitis/epidermidis* | GGCGGTTTTTTAAGTCTGATGTGAAAGCCCACGGCTCAACCGTG |
| *Staphylococcus cohnii* | GGCGGTTTCTTAAGTCTGATGTGAAAGCCCACGGCTCAACCGTG |
| *Staphylococcus haemolyticus* | GGCGGTTTTTTAAGTCTGATGTGAAAGCCCACGGCTCAACCGTG |
| *Staphylococcus hominis* | GGCGGTTTTTTAAGTCTGATGTGAAAGCCCACGGCTCAACCGTG |
| *Staphylococcus lugdunensis* | GGCGGTTTTTTAAGTCTGATGTGAAAGCCCACGGCTCAACCGTG |
| *Stenotrophomonas maltophilia* | GGTGGTCGTTTAAGTCCGTTGTGAAACCCCTGGGCTCAACCTGG |
| *Streptococcus agalactiae* | GGCGGTTCTTTAAGTCTGAAGTTAAAGGCAGTGGCTTAACCATT |
| *Streptococcus anginosus* | GGCGGTTAGAAAAGTCTGAAGTGAAAGGCAGTGGCTCAACCATT |
| *Streptococcus constellatus* | GGCGGTTAGATAAGTCTGAAGTGAAAGGCAGTGGCTCAACCATT |
| *Streptococcus dysgalactiae* | GGCGGTTCTTTAAGTCTGAAGTTAAAGGCAGTGGCTCAACCACT |
| *Streptococcus gordonii* | GGCGGTTAGATAAGTCTGAAGTTAAAGGCTGTGGCTTAACCATA |
| *Streptococcus mitis* | GGCGGTTAGATAAGTCTGAAGTTAAAGGCTGTGGCTTAACCATA |
| *Streptococcus pneumoniae* | GGCGGTTAGATAAGTCTGAAGTTAAAGGCTGTGGCTTAACCATA |
| *Streptococcus pyogenes* | GGCGGTTTTTTAAGTCTGAAGTTAAAGGCATTGGCTCAACCAAT |
| *Streptococcus salivarius* | GGCGGTTTGATAAGTCTGAAGTTAAAGGCTGTGGCTCAACTTGG |

**Supplemental Table S1 (f)**

**IMLL Q-Probe 4** (5′➞3′)

| IMLL Q-Probe 4 target sequence | GTCCACGCTGTAAACGATGAGTATTAAGAGGTTGTGCC |
| --- | --- |
| *Acinetobacter baumanii* | GTCCAＴGCCGTAAACGATGTCTACTAGCCGTTGGGGCC |
| *Acinetobacter calcoaceticus* | GTCCATGCCGTAAACGATGTCTACTAGCCGTTGGGGCC |
| *Actinomyces israelii* | GTCCATGCTGTAAACGTTGGGCACTAGGTGTGGGGGGTC |
| *Aeromonas hydrophila* | GTCCACGCCGTAAACGATGTCGATTTGGAGGCTGTGTC |
| *Arthrobacter cumminsii* | GTCCATGCCGTAAACGTTGGGCACTAGGTGTGGGGGAC |
| *Bacillus cereus* | GTCCACGCCGTAAACGATGAGTGCTAAGTGTTAGAGGG |
| *Bacteroides fragilis* | GTCCACACAGTAAACGATGAATACTCGCTGTTTGCGAT |
| *Bacteroides nordii* | GTCCACACAGTAAACGATGAATACTCGCTGTTTGCGAT |
| *Bacteroides thetaiotaomicron* | GTCCACACAGTAAACGATGAATACTCGCTGTTTGCGAT |
| *Bifidobacterium bifidum* | GTCCACGCCGTAAACGGTGGACGCTG-GATGTGGGGCA |
| *Bilophila wadsworthia* | GTCCACGCTGTAAACGATGＧGTGCTGGGTGCTGGGATG |
| *Chryseobacterium gleum* | GTCCACGCCGTAAACGATGCTAACTCGTTTTTGGTATT |
| *Citrobacter amalonaticus* | GTCCACGCCGTAAACGATGTCTATTTGGAGGTTGTGCC |
| *Citrobacter freundii* | GTCCACGCCGTAAACGATGTCGACTTGGAGGTTGTGCC |
| *Clostridium difficile* | GTCCACGCTGTAAACGATGAGTACTAGGTGTCGGGGGT |
| *Clostridium leptum* | GTCCATGCCGTAAACGATGATTACTAGGTGTGGGGGGT |
| *Corynebacterium striatum* | GTCCATGCCGTAAACGGTGGGCGCTAGGTGTAGGGGGC |
| *Eggerthella lenta* | GTCCTAGCCGTAAACGATGGATACTAGGTGTGGGGGGC |
| *Eikenella corrodens* | GTCCACGCCCTAAACGATGTCGATTA-GCTGTTGGGCA |
| *Enterobacter aerogenes* | GTCCACGCCGTAAACGATGTCGACTTGGAGGTTGTGCC |
| *Enterococcus avium* | GTCCACGCCGTAAACGATGAGTGCTAAGTGTTGGAGGG |
| *Enterococcus casseliflavus* | GTCCACGCCGTAAACGATGAGTGCTAAGTGTTGGAGGG |
| *Enterococcus faecalis* | GTCCACGCCGTAAACGATGAGTGCTAAGTGTTGGAGGG |
| *Enterococcus faecium* | GTCCACGCCGTAAACGATGAGTGCTAAGTGTTGGAGGG |
| *Escherichia albertii* | GTCCACGCCGTAAACGATGTCGACTTGGAGGTTGTGCC |
| *Escherichia coli* | GTCCACGCCGTAAACGATGTCGACTTGGAGGTTGTGCC |
| *Finegoldia magna* | GTCCACGCCGTAAACGATGAATGCTAGGTGTTGGGGGT |
| *Fusobacterium necrophorum* | GTCCACGCTGTAAACGATGATTACTAGGTGTTGGGGGT |
| *Gardnerella vaginalis* | GTCCACGCCGTAAACGGTGGACGCTG-GATGTGGGGCC |
| *Gemella morbillorum* | GTCCACGCCGTAAACGATGAGTGCTAAGTGTTGGTCTC |
| *Haemophilus influenzae* | GTCCACGCTGTAAACGCTGTCGATTTGGGGGTTGGGGT |
| *Halomonas venusta* | GTCCACGCCGTAAACGATGTCGACCAGCCGTTGGGTGCC |
| *Klebsiella oxytoca* | GTCCACGCTGTAAACGATGTCGACTTGGAGGTTGTTCC |
| *Klebsiella pneumoniae* | GTCCACGCCGTAAACGATGTCGATTTGGAGGTTGTGCC |
| *Lactobacillus crispatus* | GTCCATGCCGTAAACGATGAGTGCTAAGTGTTGGGAGG |
| *Lactobacillus jensenii* | GTCCATGCCGTAAACGATGAGTGCTAAGTGTTGGGAGG |
| *Lactococcus garvieae* | GTCCACGCCGTAAACGATGAGTGCTAGCTGTAGGGAGC |
| *Listeria monocytogenes* | GTCCACGCCGTAAACGATGAGTGCTAGCTGTAGGGAGC |
| *Morganella morganii* | GTCCACGCTGTAAACGATGTCGACTTGGAGGTTGTGCC |
| *Mycoplasma hominis* | GTCCACGCCGTAAACGATGATCATTAGTCGGTGGAGAA |
| *Nocardia cyriacigeorgica* | GTCCACGCCGTAAACGGTGGGTACTAGGTGTGGGTTTC |
| *Odoribacter splanchnicus* | GTCCACGCCGTAAACGATGCTCACTGGTTCTGTGCGAT |
| *Parvimonas micra* | GTCCACGCCGTAAACGATGAATGCTAGGTGTTGGGAGT |
| *Pasteurella multocida* | GTCCACGCTGTAAACGCTGTCGATTTGGGGATTGGGCT |
| *Prevotella corporis* | GTCCGCACGGTAAACGATGGATGCCCGCTGTTGGCGCT |
| *Prevotella intermedia* | GTCCGCACGGTAAACGATGGATGCCCGCTGTTAGCGCC |
| *Propionibacterium acnes* | GTCCACGCTGTAAACGGTGGGTACTAGGTGTGGGGTCC |
| *Proteus mirabilis* | GTCCACGCTGTAAACGATGTCGATTTAGAGGTTGTGGT |
| *Pseudomonas aeruginosa* | GTCCACGCCGTAAACGATGTCGACTAGCCGTTGGGATC |
| *Raoultella planticola* | GTCCACGCTGTAAACGATGTCGACTTGGAGGTTGTTCC |
| *Salmonella enterica* | GTCCACGCCGTAAACGATGTCTACTTGGAGGTTGTGCC |
| *Serratia marcescens* | GTCCACGCTGTAAACGATGTCGATTTGGAGGTTGTGCC |
| *Sphingomonas paucimobilis* | GTCCACGCCGTAAACGATGATAACTAGCTGTCCGGGCA |
| *Staphylococcus aureus* | GTCCACGCCGTAAACGATGAGTGCTAAGTGTTAGGGGG |
| *Staphylococcus capitis/epidermidis* | GTCCACGCCGTAAACGATGAGTGCTAAGTGTTAGGGGG |
| *Staphylococcus cohnii* | GTCCACGCCGTAAACGATGAGTGCTAAGTGTTAGGGGG |
| *Staphylococcus haemolyticus* | GTCCACGCCGTAAACGATGAGTGCTAAGTGTTAGGGGG |
| *Staphylococcus hominis* | GTCCACGCCGTAAACGATGAGTGCTAAGTGTTAGGGGG |
| *Staphylococcus lugdunensis* | GTCCACGCCGTAAACGATGAGTGCTAAGTGTTAGGGGG |
| *Stenotrophomonas maltophilia* | GTCCACGCCCTAAACGATGCGAACTG-GATGTTGGGTG |
| *Streptococcus agalactiae* | GTCCACGCCGTAAACGATGAGTGCTAGGTGTTAGGCCC |
| *Streptococcus anginosus* | GTCCACGCCGTAAACGATGAGTGCTAGGTGTTGGGCCC |
| *Streptococcus constellatus* | GTCCACGCCGTAAACGATGAGTGCTAGGTGTTAGGTCC |
| *Streptococcus dysgalactiae* | GTCCACGCCGTAAACGATGAGTGCTAGGTGTTAGGCCC |
| *Streptococcus gordonii* | GTCCACGCCGTAAACGATGAGTGCTAGGTGTTAGGCCC |
| *Streptococcus mitis* | GTCCACGCCGTAAACGATGAGTGCTAGGTGTTAGACCC |
| *Streptococcus pneumoniae* | GTCCACGCTGTAAACGATGAGTGCTAGGTGTTAGACCC |
| *Streptococcus pyogenes* | GTCCACGCCGTAAACGATGAGTGCTAGGTGTTAGGCCC |
| *Streptococcus salivarius* | GTCCACGCCGTAAACGATGAGTGCTAGGTGTTGGATCC |

**Supplemental Table S1 (g)**

**IMLL Q-Probe 5** (5′➞3′)

| IMLL Q-Probe 5 target sequence | GAACCTTACCTAATCTTGACATCCTTAGAACTTTGCAGAGAT |
| --- | --- |
| *Acinetobacter baumanii* | GAACCTTACCTGGCCTTGACATACTAGAAACTTTCCAGAGAT |
| *Acinetobacter calcoaceticus* | GAACCTTACCTGGCCTTGACATAGTAAGAACTTTCCAGAGAT |
| *Actinomyces israelii* | GAACCTTACCAGGGCTTGACATGGCCGGCTGCTCCTGGAGAC |
| *Aeromonas hydrophila* | GAACCTTACCTGGCCTTGACATGTCTGGAATCCTGTAGAGAT |
| *Arthrobacter cumminsii* | GAACCTTACCAAGGCTTGACATGGACCGGATCGGCGCAGAGAT |
| *Bacillus cereus* | GAACCTTACCAGGTCTTGACATCCTCTGAAAACCCTAGAGAT |
| *Bacteroides fragilis* | GAACCTTACCCGGGCTTAAATTGCAGTGGAATGATGTGGAAAC |
| *Bacteroides nordii* | GAACCTTACCCGGGCTTAAATTGCATTTGAATGTTGGGGAAAC |
| *Bacteroides thetaiotaomicron* | GAACCTTACCCGGGCTTAAATTGCATTTGAATATATTGGAAAC |
| *Bifidobacterium bifidum* | GAACCTTACCTGGGCTTGACATGTTCCCGACGACGCCAGAGAT |
| *Bilophila wadsworthia* | GAACCTTACCCAGGCTTGACATCTAGGGAACCCTTCGGAAAT |
| *Chryseobacterium gleum* | GAGTACGAACGCAAGTTTGAAACTCAAAGGAATTGACGGGGGC |
| *Citrobacter amalonaticus* | GAACCTTACCTGGTCTTGACATCCACAGAACTTGGCAGAGAT |
| *Citrobacter freundii* | GAACCTTACCTACTCTTGACATCCAGAGAACTTAGCAGAGAT |
| *Clostridium difficile* | GAACCTTACCTAAGCTTGACATCCCAATGACATCTCCTTAAT |
| *Clostridium leptum* | GAACCTTACCAGGTCTTGACATCCGTCTAACGAAGCAGAGAT |
| *Corynebacterium striatum* | GAACCTTACCTGGGCTTGACATATACAAGACAGGCGTAGAGAT |
| *Eggerthella lenta* | GAACCTTACCAGGGCTTGACATGGACGTGAAGCCGGGGAAAC |
| *Eikenella corrodens* | GAACCTTACCTGGTCTTGACATGTACGGAACCTTTCAGAGAC |
| *Enterobacter aerogenes* | GAACCTTACCTACTCTTGACATCCAGAGAACTTTCCAGAGAT |
| *Enterococcus avium* | GAACCTTACCAGGTCTTGACATCCTTTGACCACTCTAGAGAT |
| *Enterococcus casseliflavus* | GAACCTTACCAGGTCTTGACATCCTTTGACCACTCTAGAGAT |
| *Enterococcus faecalis* | GAACCTTACCAGGTCTTGACATCCTTTGACCACTCTAGAGAT |
| *Enterococcus faecium* | GAACCTTACCAGGTCTTGACATCCTTTGACCACTCTAGAGAT |
| *Escherichia albertii* | GAACCTTACCTGGTCTTGACATCCACGGAAGTTTTCAGAGAT |
| *Escherichia coli* | GAACCTTACCTGGTCTTGACATCCACGGAAGTTTTCAGAGAT |
| *Finegoldia magna* | GAACCTTACCAGGGCTTGACATGTGGGTGAAAGGTATAGAGAT |
| *Fusobacterium necrophorum* | GAACCTTACCAGCGTTTGACATCCTACGAACGGAGCAGAGAT |
| *Gardnerella vaginalis* | GAACCTTACCTGGGCTTGACATGTGCCTGACGACTGCAGAGAT |
| *Gemella morbillorum* | GAACCTTACCAAGTCTTGACATACTGTGAGGACACAAGAGAT |
| *Haemophilus influenzae* | GAACCTTACCTACTCTTGACATCCTAAGAAGAGCTCAGAGAT |
| *Halomonas venusta* | GAACCTTACCTACTCTTGACATCCTGCGAACTTGTGAGAGAT |
| *Klebsiella oxytoca* | GAACCTTACCTACTCTTGACATCCAGAGAACTTAGCAGAGAT |
| *Klebsiella pneumoniae* | GAACCTTACCTGGTCTTGACATCCACAGAACTTTCCAGAGAT |
| *Lactobacillus crispatus* | GAACCTTACCAGGTCTTGACATCTAGTGCCATTTGTAGAGAT |
| *Lactobacillus jensenii* | GAACCTTACCAGGTCTTGACATCCTTTGACCACCTAAGAGAT |
| *Lactococcus garvieae* | GAACCTTACCAGGTCTTGACATACTCGTGCTATCCTTAGAGAT |
| *Listeria monocytogenes* | GAACCTTACCAGGTCTTGACATCCTTTGACCACTCTGGAGAC |
| *Morganella morganii* | GAACCTTACCTACTCTTGACATCCAGAGAACTTAGCAGAGAT |
| *Mycoplasma hominis* | GAACCTTACCCACTCTTGACATCCTTGCAAAGCTATAGAGAT |
| *Nocardia cyriacigeorgica* | GAACCTTACCTGGGTTTGACATACACCGGAAACCTGCAGAGAT |
| *Odoribacter splanchnicus* | GAGCTCAACTCAATTGTGCCATTAATACTGGTAAACTGGAGT |
| *Parvimonas micra* | GAACCTTACCAAGGCTTGACATATAGTTGAGTTATTGAGAAAT |
| *Pasteurella multocida* | GAACCTTACCTACTCTTGACATCCTAAGAAGAGCTCAGAGAT |
| *Prevotella corporis* | GAACCTTACCCGGGCTTGAATTGCAGAGGAAAGATCCAGAGAT |
| *Prevotella intermedia* | GAACCTTACCCGGGCTTGAATTGCAGACGCAGGATACAGAGAT |
| *Propionibacterium acnes* | GAACCTTACCTGGGTTTGACATGGATCGGGAGTGCTCAGAGAT |
| *Proteus mirabilis* | GAACCTTACCTACTCTTGACATCCAGCGAATCCTTTAGAGAT |
| *Pseudomonas aeruginosa* | GAACCTTACCTGGCCTTGACATGCTGAGAACTTTCCAGAGAT |
| *Raoultella planticola* | GAACCTTACCTACTCTTGACATCCAGAGAACTTAGCAGAGAT |
| *Salmonella enterica* | GAACCTTACCTGGTCTTGACATCCACAGAAGAATCCAGAGAT |
| *Serratia marcescens* | GAACCTTACCTACTCTTGACATCCAGAGAACTTTCCAGAGAT |
| *Sphingomonas paucimobilis* | GAACCTTACCAGCGTTTGACATGGTAGGACGACTTCCAGAGAT |
| *Staphylococcus aureus* | GAACCTTACCAAATCTTGACATCCTTTGACAACTCTAGAGAT |
| *Staphylococcus capitis/epidermidis* | GAACCTTACCAAATCTTGACATCCTCTGACCCCTCTAGAGAT |
| *Staphylococcus cohnii* | GAACCTTACCAAATCTTGACATCCTTTGACAACTCTAGAGAT |
| *Staphylococcus haemolyticus* | GAACCTTACCAAATCTTGACATCCTTTGACAACTCTAGAGAT |
| *Staphylococcus hominis* | GAACCTTACCAAATCTTGACATCCTTTGACCCTTCTAGAGAT |
| *Staphylococcus lugdunensis* | GAACCTTACCAAATCTTGACATCCTTTGACCGCTCTAGAGAT |
| *Stenotrophomonas maltophilia* | GAACCTTACCTGGCCTTGACATGTCGAGAACTTTCCAGAGAT |
| *Streptococcus agalactiae* | GAACCTTACCAGGTCTTGACATCCTTCTGACCGGCCTAGAGAT |
| *Streptococcus anginosus* | GAACCTTACCAGGTCTTGACATCCCGATGCTATTTCTAGAGAT |
| *Streptococcus constellatus* | GAACCTTACCAGGTCTTGACATCCCTCTGACCACTCTAGAGAT |
| *Streptococcus dysgalactiae* | GAACCTTACCAGGTCTTGACATCCTCCTGACCGGTCTAGAGAT |
| *Streptococcus gordonii* | GAACCTTACCAGGTCTTGACATCCCGATGCCCGCTCTAGAGAT |
| *Streptococcus mitis* | GAACCTTACCAGGTCTTGACATCCCTCTGACCGCTCTAGAGAT |
| *Streptococcus pneumoniae* | GAACCTTACCAGGTCTTGACATCCCTCTGACCGCTCTAGAGAT |
| *Streptococcus pyogenes* | GAACCTTACCAGGTCTTGACATCCCGATGCCCGCTCTAGAGAT |
| *Streptococcus salivarius* | GAACCTTACCAGGTCTTGACATCCCGATGCTATTTCTAGAGAT |

**Supplemental Figure S1**

(b)

(a)


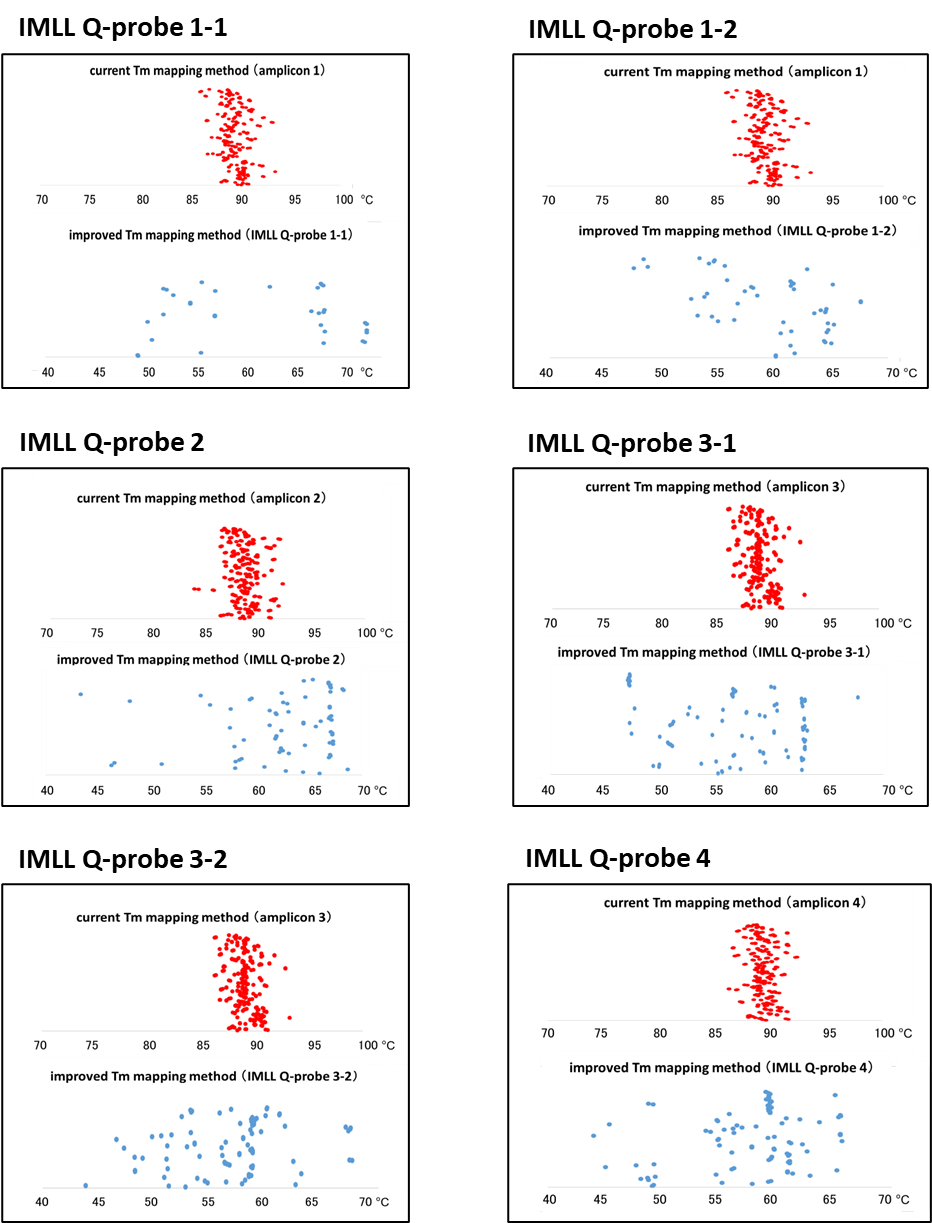


(f)

(e)

(d)

(c)

Illustrates differences between the variation range in Tm values from the current Tm mapping method and the variation range in Tm values from the improved Tm mapping method.
