# Supplementary material for: Melting temperature mapping method using imperfect-match linear long probes
Source: Sci Rep. 2024 May 14;14:11055. doi: 10.1038/s41598-024-60987-7 (PMC11094154; doi:10.1038/s41598-024-60987-7)
Supplement: Supplementary file 2 — Supplementary Information 2. [file 41598_2024_60987_MOESM2_ESM.docx]

*Supplementary Data*

**Nucleotide sequence data obtained for identification of detected bacteria shown in Table 3**

**Patient 5**

**Sequence data using Region 1 forward primer (Part 1)**

**Patient 5**

**Sequence data using Region 1 forward primer (Part 2)**

**Patient 5**

**Sequence data using Region 5 reverse primer (Part 1)**

**Patient 5**

**Sequence data using Region 5 reverse primer (Part 2)**

**Patient 6**

**Sequence data using Region 1 forward primer (Part 1)**

**Patient 6**

**Sequence data using Region 1 forward primer (Part 2)**

**Patient 6**

**Sequence data using Region 5 reverse primer (Part 1)**

**Patient 6**

**Sequence data using Region 5 reverse primer (Part 2)**

**Patient 9**

**Sequence data using Region 1 forward primer (Part 1)**

**Patient 9**

**Sequence data using Region 1 forward primer (Part 2)**

**Patient 9**

**Sequence data using Region 5 reverse primer (Part 1)**

**Patient 9**

**Sequence data using Region 5 reverse primer (Part 2)**

**Patient 15**

**Sequence data using Region 1 forward primer (Part 1)**

**Patient 15**

**Sequence data using Region 1 forward primer (Part 2)**

**Patient 15**

**Sequence data using Region 5 reverse primer (Part 1)**

**Patient 15**

**Sequence data using Region 5 reverse primer (Part 2)**

**Patient 17**

**Sequence data using Region 1 forward primer (Part 1)**

**Patient 17**

**Sequence data using Region 1 forward primer (Part 2)**

**Patient 17**

**Sequence data using Region 5 reverse primer (Part 1)**

**Patient 17**

**Sequence data using Region 5 reverse primer (Part 2)**

**Patient 18**

**Sequence data using Region 1 forward primer (Part 1)**

**Patient 18**

**Sequence data using Region 1 forward primer (Part 2)**

**Patient 18**

**Sequence data using Region 5 reverse primer (Part 1)**

**Patient 18**

**Sequence data using Region 5 reverse primer (Part 2)**
